# Integrating health care in Australia: a qualitative evaluation

**DOI:** 10.1186/s12913-019-4780-z

**Published:** 2019-12-11

**Authors:** Steven A. Trankle, Tim Usherwood, Penny Abbott, Mary Roberts, Michael Crampton, Christian M. Girgis, John Riskallah, Yashu Chang, Jaspreet Saini, Jennifer Reath

**Affiliations:** 1 School of Medicine, Department General Practice, Western Sydney University, Building 30.3.18 Campbelltown Campus, Locked Bag 1797, Penrith, NSW 2751 Australia; 2 School of Medicine, Sydney University, Sydney, Australia; 3 George Institute for Global Health, Sydney, Australia; 4 Western Sydney Local Health District (Westmead Hospital), North Parramatta, Australia; 5 Western Sydney Primary Health Network, Blacktown, Australia; 6 Royal North Shore Hospital, Leonards, Australia; 7 Western Sydney Local Health District (Blacktown Hospital), Parramatta, Australia

**Keywords:** Integrated care, Chronic illness, Qualitative, Australia

## Abstract

**Background:**

With aging populations, a growing prevalence of chronic illnesses, higher expectations for quality care and rising costs within limited health budgets, integration of healthcare is seen as a solution to these challenges. Integrated healthcare aims to overcome barriers between primary and secondary care and other disconnected patient services to improve access, continuity and quality of care. Many people in Australia are admitted to hospital for chronic illnesses that could be prevented or managed in the community. Western Sydney has high rates of diabetes, heart and respiratory diseases and the NSW State Ministry of Health has implemented key strategies through the Western Sydney Integrated Care Program (WSICP) to enhance primary care and the outcomes and experiences of patients with these illnesses.

**Methods:**

We aimed to investigate the WSICP’s effectiveness through a qualitative evaluation focused on the 10 WSICP strategies using a framework analysis. We administered 125 in-depth interviews in two rounds over 12 months with 83 participants including patients and their carers, care facilitators, hospital specialists and nurses, allied health professionals, general practitioners (GPs) and primary care nurses, and program managers. Most participants (71%) were interviewed twice. We analysed data within a framework describing how strategies were implemented and used, the experiences around these, their perceived value, facilitators and barriers, and participant-identified suggestions for improvement.

**Results:**

Care facilitators helped patients access services within the hospital and in primary care and connected general practices with hospital specialists and services. Rapid access and stabilisation clinics with their patient hotlines assisted patients and carers to self-manage chronic illness while connecting GPs to specialists through the GP support-line. Action plans from the hospital informed GPs and their shared care plans which could be accessed by other community health professionals and patients. HealthPathways provided GPs with local, evidence-based guidelines for managing patients. Difficulties persisted in effective widespread access to shared records and electronic communication across sectors.

**Conclusions:**

The combined WSICP strategies improved patient and carer experience of healthcare and capacity of GPs to provide care in the community. Information sharing required longer-term investment and support, though benefits were evident by the end of our research.

## Background

Developed countries with ageing populations face a growing prevalence of complex chronic diseases and higher expectations for quality care [1]. Health budgets are also limited and require healthcare to be delivered equitably and cost effectively [1]. Integration of healthcare has been suggested as one way of addressing these issues [2, 3]. When organisations and services work together, and where care is coordinated around the needs of people and populations, service users and their families benefit [1, 4].

Integrated care aims to link patient services by overcoming barriers between primary and secondary care, physical and mental health care, and health and social care so that patients receive comprehensive or holistic care when they need it [4, 5]. Integrated health systems aim to improve access, quality and continuity of services, especially for people with complex needs and multiple morbidity [3, 6, 7].

Integrated care models have been adopted by health systems around the world [2, 4]. Their experience demonstrates that integration is most effective when there is agreement and trust built among different providers who work together to realize a shared vision and common understanding of integrated care, through support of local service innovation [8–12]. Patient perspectives should also inform integrated care models [7]. Successful integration of health services relies on strong primary care, especially in managing those with chronic conditions [13].

Every year in Australia, more than a quarter of a million people are admitted to hospital for illnesses that could have been prevented or managed in the community [14]. Most of these are acute deterioration of patients with chronic conditions. In western Sydney, over 57% of people have at least one major risk factor for chronic illness [15]. This region demonstrates high rates of obesity, diabetes, ischaemic heart disease and respiratory diseases in comparison to other parts of Australia [16].

To address the needs of patients living in western Sydney the Western Sydney Integrated Care Program (WSICP) Demonstrator was piloted from 2014 to 2017 [17, 18]. This model had four aims [17, 18]:

1. improve the health of patients with chronic illness;
2. enhance patient experience;
3. reduce costs of health care; and
4. better support health professionals in caring for these patients.

The WSICP aimed to improve the management of chronic conditions in primary care and strengthen the Patient Centred Medical Home (PCMH) model being implemented in general practices in western Sydney. The PCMH model of care focuses on improving primary care and the healthcare experiences of a registered patient cohort [19]. The principles of a PCMH in Australia include improved access to whole person, patient-centred care through a team-based approach; care coordination and/or integration; quality and safety improvements to support evidence-based medicine; and enhanced care availability including extended hours and non-face to face care options [20].

Using evidence based approaches to integrating healthcare [4, 21–23], the Western Sydney Local Health District (WSLHD) worked in partnership with the Western Sydney Primary Health Network (WSPHN) to develop and implement the WSICP as an innovative, integrated service model for co-ordinating care for those experiencing any of the three priority chronic diseases in western Sydney - diabetes, congestive cardiac failure (CCF) and chronic obstructive pulmonary disease (COPD) [15].

The WSICP engaged healthcare professionals in two key WSLHD hospitals who were providing care in the prioritised chronic disease areas. The WSPHN and WSICP hospital staff enrolled general practices in the region. Patients and carers were enrolled in the program by WSICP staff during hospital visits, and by engaged general practices.

The WSICP provided a range of strategies designed to enhance primary care capacity, strengthen partnerships and integration between service providers, and develop and implement new shared care protocols [17] (Table 1).

**Table 1:** WSICP Strategies

WSICP Strategies	Description
Care Facilitators	Employed by the WSLHD to liaise between patient, general practice, hospital and other services to assist in supporting and coordinating services for patients
Information Technology (IT)	Initiatives to improve sharing of information between hospital and community sectors
Shared Care Plans	Developed in general practices and shared with hospitals, community health providers and patients via a “Linked Electronic Health Record (EHR)”
Specialist Action Plans	Provided at hospital discharge and intended to inform patients and general practitioners about care in complex situations and where treatment is changing frequently
GP Support Line	Provides General Practitioners (GPs) with faster access to hospital specialists including referral to rapid access clinics as required
Rapid Access and Stabilisation Service (RASS) Clinics	Provided to reduce unnecessary hospital admission and re-admission and including Patient Hotlines to facilitate patient access to the clinics
HealthPathways	Online access for GPs to referral and other health service information
Support Payments to General Practices	To facilitate patient enrolment and care planning
Promotion of Patient Centred Medical Home	Support for general practices to lead multidisciplinary teams that provide comprehensive coordinated care especially by improving efficiency and use of information technology. Training to expand staff roles in practices.
Communication between WSICP and non-WSICP Services	Connecting better between hospital and other government and non-government services for patient needs.

## Research aims

We aimed to understand how the WSICP was working, including its facilitators and barriers, from the perspectives and experiences of the different stakeholders engaged in this integrated health care initiative. Our research complemented quantitative data collection and analysis conducted by the NSW Ministry of Health. In this article we report the findings of a qualitative evaluation of the implementation of the various WSICP strategies within the context of the wider evaluation which is reported elsewhere [24].

## Methods

### Design of the study

We conducted a qualitative evaluation using in-depth interviews to explore the experience and satisfaction of participants with each of the WSICP strategies and processes [25, 26]. Interviews were undertaken in two rounds up to 12 months apart to collect information on changes over time. Our reporting of qualitative data aligns with the COREQ guide [27] (Additional file 1).

### The researchers and WSICP evaluation management

The primary research team included qualitative researchers from Western Sydney University (WSU) and University of Sydney with extensive experience in health services research. The research manager (ST) was an experienced interviewer. Co-investigators from the Western Sydney Local Health District and Western Sydney Primary Health Network were western Sydney clinicians who advised the research team particularly concerning recruitment and assisted in interpreting interview data.

Throughout the evaluation, we reported progress to the Integrated Care Evaluation Advisory Committee. This committee, consisting of clinical and administrative leaders and a consumer representative, was responsible for overseeing WSICP implementation and the many related evaluation activities.

### Sampling and recruitment

We consulted with the Integrated Care Evaluation Advisory Committee and agreed on a sampling frame of key stakeholder groups who would provide diverse perspectives on the WSICP. These included patients and carers enrolled in the WSICP, management staff of the WSICP, a range of clinical and allied healthcare providers from Westmead and Blacktown hospitals, and general practitioners (GPs) and staff from participating general practices in western Sydney. We stratified our sampling frame across the stakeholder groups and across the three chronic illness areas of diabetes, COPD and CCF.

Patients and carers were provided with information about the study and recruited opportunistically during their hospital appointments by the co-investigators and by care facilitators who recruited from general practices. Contact details of consenting patients and carers were given to the research manager (ST). Hospital clinicians were purposively sampled to include a range of healthcare providers working with the WSICP. These participant groups were told about the study by WSICP program managers and clinical co-investigators at Blacktown and Westmead hospitals, and consented to their contact details being mailed to the Western Sydney University research manager. Management staff volunteered to participate directly with the researchers. General practitioners and their nursing staff who were enrolled in the WSICP were recruited by the Western Sydney Primary Health Network staff who similarly provided them with information about the study and, with their verbal consent, mailed contact details to ST. All those referred to ST provided written informed consent when contacted to schedule their interview or at the time of interview. Most participants did not know the researchers prior to the study commencing. Although many GPs were known to the researchers, they were not known to the research manager prior to the study and their data were de-identified following transcription of the interviews.

We continued recruiting until our sample approximated the targeted total of 70 participants. This sample ensured all stakeholder groups were adequately represented. Participants confirmed their contact details and signed a pre-consent form for a subsequent interview. We also recruited some new patients and carers and staff from general practices in the second round of interviews using the same process as the first round. No participants referred to ST declined participation, however, not all participants were available for a second interview.

### Interviews (data collection)

We examined the literature and consulted with the Integrated Care Evaluation Advisory Committee to design a semi–structured interview guide for collecting participant perspectives and experiences on the WSICP strategies and their implementation. The development of initial interview questions was guided by program management, clinical leaders and a consumer representative to ensure their relevance for these stakeholder groups and included some common questions. The guide was refined by the primary research team in meetings with program managers and clinical co-investigators. We piloted the guide in the first 10 interviews and then reviewed the interview transcripts within the research team to ensure the clarity and comprehensiveness of the interview questions. Informed by our ongoing data analysis throughout the data collection period, we continued to revise the guide as necessary by adding questions and prompts to explore the emerging areas of interest in more depth. Before we began the second round of interviews, we refined the interview guide again in consultation with the research team and the co-investigators. This ensured important issues arising from the first round could be included as well as questions about changes over time (Additional file 2). In each of the interview rounds the earlier interviews often informed questions for later interviews which helped us reach data saturation.

All interviews were conducted one-on-one by a single interviewer (ST), mostly face to face in hospital and general practice offices although some (40%) were conducted by telephone according to participant preference. Interviews were between 30 and 60 min in duration, audio-recorded and then transcribed by an independent transcription service. Transcripts were checked for accuracy and interviewees were offered the opportunity to review their transcript.

### Analysis

We analysed data in both interview rounds using a framework corresponding with each of the WSICP strategies. The framework method of qualitative analysis is a systematic and flexible approach to data analysis which supports a holistic, comprehensive and descriptive overview of an extensive dataset [28–30].

We developed the framework matrix a-priori in consultation with the research team and our clinical co-investigators to capture specific aspects of interest related to each of the WSICP strategies and their implementation. We systematically organised our data into codes and categories within the agreed matrix to describe how strategies were implemented and used, the experiences around these, their perceived value, facilitators and barriers in achieving the desired program outcomes, and participant-identified suggestions for the future. We used N-Vivo 11® software to help organise our data coding.

The initial 10 interview transcripts were coded independently by four research team members (ST, JR, TU, PA) and one clinical co-investigator (MR) who each coded up to three transcripts, with some cross-coded between members. We then met to discuss our coding and examine alignment with the framework. The framework comprehensively captured the data and did not need further revision. ST then conducted the remainder of the interviews and coded these. Due to the large number of transcripts, we checked coding in an iterative process through ongoing discussion of the analysis in a series of research team meetings. Upon completion of coding, the primary research team met to review the completed coding matrix and discuss the positioning of individual categories and codes and ensure the framework remained accurate and inclusive of the data.

In order to further ensure the veracity of the data, we reviewed our analysis with the Evaluation Advisory Committee of the WSICP at the end of each of the two interview rounds.

## Results

We conducted 125 interviews in total with 59 people in the first interview round between March and September 2016, and 66 in the second round from late November 2016 to March 2017 (Table 2). We interviewed 12 patients enrolled in WSICP, with 8 patients participating in both the first and second round of interviews, and 11 carers. Twenty-nine Western Sydney Local Health District employed healthcare providers participated in the evaluation, including medical specialists, registrars, nurses, allied healthcare providers and WSICP care facilitators. The majority of these (71%) took part in both interview rounds. Twenty-one GPs and general practice nursing staff participated, with 38% of this group participating in both rounds. Additionally, 10 members of the Evaluation Advisory Committee comprising a mix of managers and clinicians with advisory roles were interviewed in one or both rounds. Participants were spread almost equally across the three chronic disease areas and were from both Westmead and Blacktown hospital catchments. General practice participants were recruited from 14 different practices across western Sydney.

**Table 2:** Participants

Participant Group		First Round	Second Round	Total participants^a^
Patients/carers	*Patients*	11	9	12
	*Carers*	1	10	11
	Total Patients /Carers	12	19	23
Healthcare providers	*Hospital specialists and registrars*	12	8	12
	*Hospital Nursing staff*	7	6	7
	*Hospital Allied Healthcare Providers*	5	4	6
	*General Practitioners*	7	12	14
	*General Practice Nurses*	3	7	7
	*Care Facilitators*	3	3	4
	Total Healthcare Providers	37	40	50
Evaluation Advisory Committee (Titled “managers” in our results)		10	7	10
Total Participants		59	66	83

^a^Excluding duplications across rounds 1 and 2

We examined how each of the 10 WSICP strategies listed above (Table 1) were implemented and utilised, how participants experienced and valued these, the facilitators and barriers in realising the desired program outcomes, and suggestions for the future as identified by participants. We noted that positive experience, satisfaction and perceived value often overlapped. Similarly, negative experience was often linked with suggestions for system improvement. The larger analysis table with all quotations is available as “Additional file 3”.

***Care facilitators*** were said to help patients by providing information both about their health conditions, following their care plans and navigating health services, especially through support to attend appointments. They were a primary contact for patients and important patient advocates as well as familiar faces in the hospital.

*… having an advocate, having an educator or someone with them, has a big impact on definitely service navigation and the understanding of their chronic disease (Care Facilitator 3 Round2).*

Care facilitators often recruited patients and GPs into the program and were considered a crucial link between hospitals and community based care, following up transitioning patients and providing information about patient progress to healthcare providers in both sectors. Care facilitators often linked patients in with services beyond those provided by the WSICP. The care facilitator role was reported to be critical in improving integrated care, and the knowledge and skills of those in these roles were highly regarded by our interviewees.

*… having [Care Facilitator] look at the patient’s care plan and also them having done an assessment of the patient and that assessment being communicated to the GP, has been particularly valuable … we’ve picked up extra issues that we weren’t aware of (General Practitioner1 Round1).*

However, care facilitators reported a heavy workload and limited information and support for their roles initially. Their job descriptions were unclear, especially in the early stages of the WSICP. This appeared to have improved by the second round of interviews.

***Information technology (IT) systems*** were reported by the healthcare providers we interviewed to be very frustrating. They noted poor functionality of shared health records, and minimal IT enhanced communication between hospitals and community sectors. In the absence of IT solutions, recourse to traditional communications such as letters, faxes and phone calls was described (and sometimes preferred) and email was used in place of shared records. Some interviewees suggested that IT services had been under-resourced, particularly the shared electronic health record (Linked-EHR).

*… it’s not working at the hospital end yet and it’s not working at the majority of the GP practices as well, so I’m not sure if Linked-EHR is really worth it … feedback that I get from GPs, after two attempts they’re like, “I don’t want to do this. I don’t even know if it’s worth it” (Care Facilitator 3 Round1).*

Training in use of IT was also reported to be inadequate and this was perceived to impact negatively on GP engagement in WSICP, especially in less IT-enabled general practices. In the hospital setting, shared care plans were often inaccessible and interviewees expressed frustration with the hospital IT system, the need to duplicate data entry, and difficulties linking with multiple different GP systems. Common recommendations from interviewees included training in use of IT and establishing an interactive common shared health record with immediate access to shared information and alerts when providers entered updates.

Despite the challenges, WSICP was seen by some interviewees to be leading in the use of IT to overcome barriers to integrated care. They recognised the future potential for the IT initiatives being pioneered through WSICP including in data collection for ongoing evaluation of the program. General practice staff valued improved communication with hospitals including at discharge, though this was not completely achieved by the second round of our interviews.

*… online documentation has been pretty good … typing straight into notes … the way of the future rather than having a big set of medical notes; just for everything to be online and integrated care is a bit of a pioneer leading the way (Allied Health 2 Round1).*

*I’m … learning about patients being in hospital much sooner. And therefore I’m able to follow up those patients a lot sooner as well (General Practitioner 9 Round2).*

***Shared patient care plans*** were developed by general practitioners to enhance communication between hospital and community based healthcare providers and also with patients and carers. The template provided by WSICP on the HealthPathways website was praised by practice nurses as easier to use than the template used prior to WSICP. The Primary Health Network provided assistance to set up the care plans in practices and both GPs and patients valued the improved efficiency from sharing care plans especially through greater connectedness with allied healthcare providers. Patients and carers described a sense of patient centredness with care plans focused on their needs and providing them with greater control.

*I do really like the ability to use the care plans or to share the care plans with allied health professionals (General Practitioner 2 Round1).*

*… all the focus is on the patient, now he directs the care plan (Patient/Carer 18 Round 2).*

However, some GPs and allied healthcare providers were not using the on-line shared care plans, preferring to e-mail and then later upload reports manually to the care plan. General practitioners were also concerned about complexity and the time needed to upload care plans and, although care facilitators sometimes assisted them with this process, they strongly recommended a more streamlined procedure.

Hospital staff could not access the care plans so care facilitators provided a summarised version in hospital records. This also meant that hospital staff could not provide feedback to GPs on the care plans. The intention for shared patient care plans to improve cross sectorial communication was therefore not fully realized by the second round of interviews.

***Specialist action plans*** were described as a useful line of communication, helping to guide more complex patient management especially in situations where treatment needed constant review. Interviewees noted actions plans were included in discharge letters handed to the patient and sent electronically or mailed to the GP and some recommended sharing these through Linked-EHR. General practitioners valued the advice action plans contained but they were not always received and sometimes required general practice staff to follow up with the hospital to access these.

Hospital based interviewees described a team based approach to the specialist action plan and were keen to hear feedback on how these plans were working for GPs. They noted that action plans were time consuming to prepare but general practitioners and patients valued the plans as being targeted to patient needs. Although patients felt reassured and found them helpful in self-managing their illness, general practitioners asked for action plans to be simple and user friendly.

*… the letter they gave us when I brought [husband] home reassured me in that I was already doing the right things and helped me so much in planning for other things … (Patient/Carer 22 Round2).*

*Make it as basic as possible. Saying, are you short of breath, take your Ventolin. If you start to cough up green stuff, take this and make sure that that plan is given to the patient, is given to the GP as well so everyone knows what the plan is (General Practitioner 1 Round1).*

***The GP support line*** commonly referred to as the “GP Hotline”, connected GPs with hospital specialists for clinical information and patient referral to rapid access and stabilisation including outside normal hours, and avoided the usual emergency department admission pathway.

Initially, GPs and hospital staff had little awareness of the service. Although this improved by the second interview round, the support line was still perceived to be underutilised and interviewees recommended advertising this WSICP initiative more effectively. Those GPs using the line, and the care facilitators, found the service helpful and a good way of building capacity to manage illnesses in the community. The support line was valued as an important connection with specialists even in situations where patients were not enrolled in the WSICP, and emergency department (ED) staff were also using the support line.

*GP … had a question about someone with lack of thyroid … it’s a really, really good idea, again, empowering the GP … building their skills … access to endocrinologist (Allied Health 2 Round1).*

***Rapid access and stabilisation service (RASS) clinics*** **Rapid Access clinics** were developed for patients needing immediate specialist review so they could bypass ED and avoid admission. **Stabilisation clinics** provided patients with post admission care and the possibility for earlier release from hospital. In the early stages, most referrals to rapid access came from the Emergency Department (ED) rather than GPs. Initial communication problems between GPs and the RASS clinics were overcome as use of GP support line and specialist action plans increased, and care facilitators also helped RASS staff to follow up patients. By the second interview round, more GPs reported that their patients were accessing specialist care quickly, and patients were excited about being “fast tracked” for care and avoiding admission. They valued being attended to by familiar staff who provided them with holistic care and information helping them to self-manage their illness and, at times, home care was also provided especially as some patients were too ill to attend hospital.

*… we do a lot of outreach visits to patients … the aim is to provide patient care at their home, because these patients are just too breathless to worry about coming in to hospital (Hospital Nurse 3 Round2).*

Hospital staff valued the ongoing and comprehensive, team based care and noted increased referral to the RASS from within the hospital. The RASS care team became familiar to patients and their carers. The clinics also linked patients from existing non-WSICP services and at times the care was reported to extend across multiple specialty areas.

*…see them with the cardiovascular team and the foot team…improved outcome if we improve their blood sugar levels…that’s one of the examples of rapid access which can actually be quite transformative (Hospital Specialist 5 Round1).*

General Practitioners described rapid access as particularly helpful for patients who had complex illnesses or were constrained financially. They praised the RASS as an effective option for patient referral rather than the ED while noting challenges with exclusionary criteria for some patients, such as those with co-morbid mental health conditions, who were excluded. However, others suggested that some patients may see rapid access as an easier way of receiving treatment than visiting their GP and therefore potentially jeopardising relationships between patients and their GPs.

*The clinics have been useful…quick access for the patients to be seen…not have to just go to ED…a step in between now and that’s really important (General Practitioner 6 Round1).*

*…they get breathless, or they are sick for a couple of days – they don’t go to their GP, because…my GP is too busy, or they are just too breathless to make the trip…the easiest thing for them to do is call the ambulance (Hospital Nurse 3 Round2).*

Stabilisation clinics providing effective post-admission care were valued for preventing re-admission and some interviewees recommended expanding the capacity of the RASS clinics to include additional areas such as chest pain and arrhythmia.

**The patient hotline** was an initiative that grew from the RASS clinics to help patients more easily contact their care team in the clinics. Patients valued this dedicated point of extended hours contact with someone familiar especially when their GP was unavailable. This connection with patients was also highly regarded by hospital staff who described it as a “safety net”, and GPs reported their patients were reassured and could better self-manage their condition with information received through the hotline. Although this was not a planned WSICP initiative, many interviewees strongly recommended continuing and promoting this service.

*if anything happens to you, no matter what time of the day it is, give them a phone call. They pride themselves on a 100% pickup (Patient/Carer 2 Round1).*

*I think it improves the patient’s understanding of how to manage their condition, plus giving them extra education…gives that patient extra reassurance and autonomy as well (General Practitioner 6 Round1).*

***HealthPathways*** was described as a streamlined online process for GPs to access treatment guidelines as well as referral and other health service information. General practitioners used it as a local and up to date guide to best practice, and regarded it important for improving their knowledge. Younger GPs were said to use HealthPathways more often, but it was also helpful for more experienced GPs in referring correctly to different hospital services. Practice nurses and care facilitators also reported using HealthPathways and sometimes provided GPs with the information to help plan investigations and management of patients.

Whilst some interviewees found it easy to access and navigate the site others described the time needed to explore all the information challenging, and particularly when doing this within the consultation. Hospital specialists reported having devoted much time in writing and updating HealthPathways. Greater promotion and expansion of this resource were repeatedly recommended.

*…when I do use it, it’s extremely useful but it’s just having that time to access it and just remember it’s there…I’ve gone back and looked at it a few times after a patient’s gone (General Practitioner 9 Round2).*

***General practice support payments*** were provided to encourage WSICP patient enrolment but not all the GPs and practice staff we interviewed understood the purpose of the payments. Most agreed that this one off payment, whilst appreciated, did not adequately cover their costs of participating in WSICP and payments were reported not to be a motivating factor for engaging with the program. Practice staff noted the need for funding patient follow up.

*I know the practice gets something but we do so much more work-identifying and enrolling patients, preparing care plans, follow ups and things like that (Practice Nurse 7 Round2).*

***The patient centred medical home (PCMH)*** was described by interviewees as a local initiative developed through the Primary Health Network and strongly supported by Local Health District stakeholders, particularly as a way of supporting care in the community. Practices transitioning to a Patient Centred Medical Home model of care were said to have stronger engagement with WSICP and, general practitioners, especially those involved in the initiative, valued the team based approach to holistic, patient-centred care. Some interviewees reported insufficient funding of this initiative which was also seen as more comprehensive than the narrow disease-specific focus of WSICP.

*There’s not enough funding there. And it’s too restricted to just a certain cohort of patients. So we are just pressing on with developing a PCMH-style practice, with the current funding model, and just figuring out how to best use what we have… (General Practitioner 7 Round2).*

***Communication with other (non-WSICP) services*** was reported as increasing, particularly in the later interviews. Recognising the need to work with others outside the program, interviewees described the use of portals such as My Aged Care, and new and growing connections with other Western Sydney Local Health District services, private healthcare providers, community based allied healthcare, and other government and non-government agencies. General practitioners referred to expansion of their care teams to include pharmacists. Particularly in prevention and management of diabetes, WSICP was seen to be working with services beyond the health sector.

*…doing a bit of that work in diabetes, which is about urban design, transport, food supply and physical activity (Hospital Specialist 8/Manager 8 Round1).*

Patients valued the in-home care they could access through some of these agencies. Healthcare providers reported being more aware of community services but also described problems with communication. Interviewees recommended improving access to information about these services and also co-locating hospital staff within the community.

*My doctor organised a community nurse who calls in regularly-because I can’t get out easily with my COPD-she checks up on me and lets me know about transport and things like that (Patient/Carer 2 Round2).*

## Discussion

The following discussion summarises our key findings and considers the WSICP and its strategies in relation to the literature. We then consider the contribution of our findings beyond the local context.

We conducted our qualitative evaluation of the program over a 12-month period from March 2016. This allowed us to gather information on each of the WSICP strategies from a range of participants at early and later stages of implementation. Time is needed to develop trust, understanding and new ways of working together in order to embed integrated care and scale it across the health system [4] and, considering the relatively short 3-year time frame of WSICP operations, there was evidence of significant achievement in relation to each of the WSICP strategies. We found care facilitators helped connect patients and general practitioners (GPs) with services and were a source of patient education. They were also a conduit for information between GPs and hospital. Effective information technology (IT) is crucial for sharing information between GPs, hospital healthcare providers and patients and was improving toward the end of our evaluation. Strategies like GP care plans and specialist action plans became important sources of information for hospital and community healthcare providers and for patients but were restrained by IT capacity. The GP support line was valued by GPs as a means of accessing advice from hospital specialists and its use was increasing over the course of our evaluation. The Rapid Access and Stabilisation clinics provided patients and referring GPs with fast access to specialist services and reduced the need for hospital admission and readmission. HealthPathways was valued by GPs as a source of current medical and referral information. General practices received support payments for enrolling in the program but considered these token and not a motivation for their engagement. Interviewees noted many parallels between the WSICP and Patient Centred Medical Home initiatives, and strengthening of collaborations with non-WSICP service providers.

Whilst the WSICP aims were similar to the aims of other integrated care programs, there were commonalities and differences between the WSICP strategies and those implemented in other programs. Indeed, care facilitation and coordination, a critical WSICP strategy, has been found effective in integrating care in other primary health contexts and reducing hospital readmission for chronic illness [31–33]. A recent Australian study similarly described a reduction in hospital admissions associated with use of general practice liaison nurse care coordination for elderly patients with complex needs [34].

The importance of sharing information between health sectors and with patients is also well recognised [35] and the WSICP focussed strongly on connecting primary and secondary care practitioners and informing patients. Shared care plans in community care and action plans from hospital specialists are considered effective [36, 37] and were central strategies for enhancing communication in the WSICP. However, challenges with information technology reduced the impact of these strategies and these problems persisted through our evaluation. Information technology whilst a key enabler of shared information [38, 39], is a common source of frustration in integrating health care [40–42] and in Australia there is a lack of effective information sharing between and within health sectors [34]. Toward the end of the evaluation some action plans were being e-mailed with discharge summaries to GPs, however, the predominant form of delivery was still hard copy either via the patient, or through facsimile or regular paper mail. This caused delays and often required follow up. Care facilitators became the informational link between hospital and GP. Hospital and general practice staff alike recommended implementing a single and widely shared electronic health record where shared care and action plans could be accessed by all healthcare providers in the community and in the hospital as well as by patients. Although Linked-EHR was meant to be the platform for sharing this information, its implementation and use in the WSICP was incomplete by the end of our evaluation. Digital technologies provide the potential for this comprehensive, real time access and can facilitate prevention, management and coordination of services [43].

Rapid access clinics are noted for reducing hospital admission rates and length of stay for cancer patients through provision of more timely specialist interventions [44]. The WSICP Rapid Access and Stabilisation clinics aimed to provide a multidisciplinary team based approach to each of the targeted chronic illnesses. These clinics provided 15,085 occasions of service between July 2015 and July 2017 and had contributed to a reduction in hospital admission and length of stay for these patients [45]. Specialist telephone support for primary care doctors can also increase the capacity for managing illness within the community and reduce unnecessary outpatient and emergency department visits by patients [46]. The WSICP GP support line provided GPs with much valued 24-h access to specialist cardiac, respiratory and endocrine teams in the Rapid Access and Stabilisation clinics to assist them in management of enrolled and also non-enrolled patients. From September 2015 to June 2017 there were 545 calls from GPs to the Rapid Access and Stabilisation specialists [45]. Initially these clinics and support line facilitated greater integration within the hospital but integration became more widespread as GPs increased their use of these services. At the end of our evaluation (June 2017), 208 GPs were registered with the WSICP and referred 1428 of the total 1510 patients enrolled in the program [45].

Strategies such as HealthPathways have improved integration in other health systems [39] and likewise provided WSICP enrolled GPs with up to date, localised referral information. The management of WSICP also recognised that holistic and integrated care needs to include services beyond those focussed on health care [47, 48] and the program was observed to build partnerships with providers outside the program in order to access services needed by their patients. These included linking with other government and non-government organisations to deliver services such as transport and in-home care as well as liaison with community-based medical specialists and allied health professionals.

A defining feature of this integration model was the focus on enhancing primary healthcare, in alignment with the identified importance of a strong and well supported primary healthcare sector in integrating patient care needs and delivering better patient outcomes [49]. Our participants reported increasing awareness and use of HealthPathways and the GP support line as the program became more established. These strategies provided GPs with access to evidence based guidelines and contact with Rapid Access and Stabilisation clinic specialists. Specialist action plans from the patient’s multidisciplinary hospital team further informed GPs. General practice respondents described learning through these approaches and increasing their capacity to manage their patients’ chronic illnesses more effectively. Other primary healthcare providers in the community also benefitted from WSICP strategies with GP shared care plans highly valued and more readily accessed including by allied healthcare providers as well as by patients. It was unfortunate that hospital staff were not able to access these as easily.

Importantly, integrated patient-centred care can improve patient understanding and enhance self-management [50]. Personalised strategies can also create an environment fostering patient self-determination [51] and our evaluation described patient empowerment resulting from their engagement in the program. Accessing care for complex illness can be difficult though [52], and in the WSICP model care facilitators connected patients with required services and provided information to assist patients in better managing their own illnesses. Patients also received education from hospital staff in the Rapid Access and Stabilisation clinics and from GPs. The action plans from the hospital and the care plans issued by GPs were specific to the patient’s needs and ongoing illness management. Patients reported greater knowledge of how to access the services they needed and who to contact when they wanted help. A “patient hotline” evolved out of the Rapid Access and Stabilisation clinics enabling patients to contact a clinic nurse or specialist directly at any hour of the day to receive advice. All patients and carers valued the holistic care and support they received through the WSICP, describing their improved ability to self-manage chronic illness with fewer admissions to hospital.

Our evaluation has described the experiences and processes involved in implementing an integrated care model which may inform similar strategies elsewhere given that effective management of chronic illness is a national priority [53] and a problem for health systems internationally [54, 55]. Of particular importance was building capacity in primary healthcare. Where this has occurred elsewhere, improved outcomes have been observed [56]. Greater coordination and integration might be better achieved by locating hospital staff like care facilitators in the community where their focus is on helping patients stay out of hospital. This could further strengthen primary care. It is also important to promote integration strategies such as the GP support line and Rapid Access and Stabilisation clinics as these were found effective in linking GPs with hospital specialists to receive advice on illness management and referral as well as in providing patient centred, ongoing specialist care. Effective care integration is also reliant upon fully functional information technology systems that can facilitate access to shared care planning. The WSICP highlighted the effectiveness of a patient centred-approach which created an environment for self-management and empowerment. Whilst other programs have used similar initiatives to WSICP, the comprehensive suite of strategies appear to have contributed to the particular success of WSICP and may also be useful in other countries.

## Strengths and limitations

The strengths of this research lie in our comprehensive evaluation of each of the WSICP strategies, informed by a large number of interviews with a wide range of stakeholders. We also collected longitudinal information to understand program changes over time. The research findings have informed ongoing implementation of WSICP strategies. At a time that health systems around the world are moving towards greater integration, our findings also provide insight to the effectiveness of the various strategies used in the WSICP.

Although we noted increasing momentum with strategy implementation and engagement during the course of the evaluation, not all participants, particularly patients/carers and GPs, joined the program at the same time. Those engaging later may have had different experiences of the program. We were also limited in gathering longitudinal information from more recent participants due to the timing of their engagement in the program. Further evaluation at a later stage, including with later participants, may assist in assessing the value and sustainability of the program and could identify additional recommendations for improving the WSICP. This could involve continuing qualitative data collection and linking with hospital statistical data, particularly admission and readmission rates, GP and patient enrolments to the program, and patient clinical outcome measures. Another helpful way of demonstrating WSICP outcomes could be to map individual patients and carers’ journeys and assess the changes in their experiences [57].

## Conclusion

In its 3 years of operation, the WSICP successfully implemented a suite of strategies which were improving management in three chronic disease areas. The combination of WSICP strategies was particularly effective for enhancing patient and provider experiences, supporting and building primary care capacity and improving patient access to hospital services while reducing the need for hospital admission. Patients were increasingly empowered to self-manage their illnesses and access timely and appropriate care. Care facilitators, Rapid Access and Stabilisation clinic staff and GPs provided educational information that focused on illness prevention and management. General practitioners increased their skills through their strengthened collaboration with hospital specialists. However, the success of integrated care programs such as the WSICP rely on effective information sharing across healthcare sectors. While this was improving in the WSICP over the course of our evaluation, difficulties persisted in widespread and seamless access to shared records and to electronic communication across sectors. Key learnings for sustained and wider healthcare integration include maintaining the care facilitator role, continued enhancement of primary healthcare, promotion of WSICP strategies such as the GP support line and Rapid Access and Stabilisation clinics, and developing information technology capacity for effective sharing of information.

## Supplementary information

**Additional file 1.** COREQ (COnsolidated criteria for REporting Qualitative research) Checklist.

**Additional file 2.** Interview Schedules (WSICP Qualitative Evaluation).

**Additional file 3.** Matrix coding of specific WSICP initiatives with illustrative quotations.

## Data Availability

Raw data is not available for public access due to ethics requirements of privacy. The authors declare that de-identified data supporting the findings of this study are available within the article and an additional file.

## Abbreviations

CCF: Congestive cardiac failure
COPD: Chronic obstructive pulmonary disease
COREQ: Consolidated criteria for reporting qualitative research
ED: Emergency Department
GP: General Practitioner
IT: Information Technology
Linked EHR: Linked Electronic Health Record
PCMH: Patient Centred Medical Home
RASS: Rapid access and stabilisation
WSICP: Western Sydney Integrated Care Program
WSLHD: Western Sydney Local Health District
WSPHN: Western Sydney Primary Health Network
WSU: Western Sydney University
