# Supplementary material for: Integrating health care in Australia: a qualitative evaluation
Source: BMC Health Serv Res. 2019 Dec 11;19:954. doi: 10.1186/s12913-019-4780-z (PMC6907151; doi:10.1186/s12913-019-4780-z)
Supplement: Supplementary file 2 — Additional file 2. Interview Schedules (WSICP Qualitative Evaluation). [file 12913_2019_4780_MOESM2_ESM.docx]

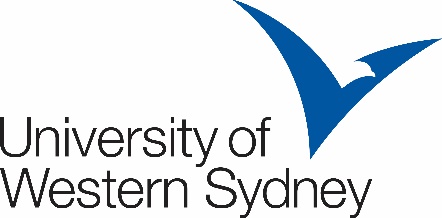
 [
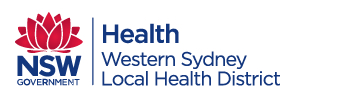
](http://wslhdold.staging.elcomcms.com/)

**Interview Schedules (WSICP Qualitative Evaluation) ROUND 1**

**Interview Questions for Patient/Carer**

**1. What sorts of health issues do you have now? (Or have had over the last five years)?**

a.       What have been your experiences in managing your health issues in the past?

b.       What are the most important things that need to happen to keep your health on track?

**2. Were you getting the health care you needed before the WSICP started?**

a.       Had you been accessing health care services through your GP? What services were these?

b.       Had you been accessing services at the hospital? What services did you access?

c. Had you been accessing community based services outside the hospital or GP setting?

What services were these?

d.       In what way have doctors and nurses and other health care providers been involved in your

health care?

e.       How has the healthcare system been working for you?

                       i.      Can you give me some examples of positive experiences you had before the WSICP

started?

                       ii.      Can you give me examples of negative experiences you had before the WSICP

started?

**3.       What was it like taking part in the WSIC program?**

a. How did you hear about it?

b.       What services did you have access to?

c.      In what way have your experiences of health care changed compared to your previous

experiences?

d. What can you say about support you received after joining the WSICP?

e.       Which components of the WSICP were most helpful to you - in improving your health?

f.      What do you think could be improved?

**4.       Could you please tell me about your experience of the following (if applicable)?** *(For each point explore how these helped or didn’t help):*

a.    Being allocated a Care Facilitator?

b. The Relationship Manager (or GPLN when role confirmed)

c. The rapid access hospital based service?

d.    A shared care plan?

**5.       What role did your health care provider(s) play in the WSICP?**

a.       Were there any changes in the way your GP managed your care while taking part in the

WSICP?

                          i.      What were they? Can you give me an example?

                          ii.      Were there any changes in the way the general practice you attend was set up?

iii. When you went to see the GP were there any changes in the way your visit there

proceeded?

iv. Did you spend more time with other staff in the practice e.g. practice nurses than

you did before?

v. How did community health services assist in your health care?

b.      Do you feel that the GP and other practice staff communicated well with you and with

others outside the general practice (e.g. hospital and community HCPs)?

                           i.      Do you think the WSICP program led to any improvements in communication

between GPs and hospitals as regards your own health care? (E.g. through

shared care plan and rapid access service).

ii. What improvements have you seen in communication with community based

services for your own health care?

c.      Have you got any views on how this program may have assisted your GP? / Your hospital

nurses and doctors?

**6. How did your experience in the program affect your ability to:**

a.      get the care you need from the health system?

b.      Manage your own health condition

**7. What have you learned:**

a.      About yourself/your health?

b. About getting the care you need from the system

c. Are there better ways you could get the health care you need?

Please explain.

**8.      What challenges do you still encounter or believe that you will continue to encounter?**

a.       Access

b.      Waiting times

c. Cost

d. Transport and parking

e. Communication and spending sufficient time with you

**(WSICP Qualitative Evaluation)**

**Interview Questions for Service Providers and Advisory/Working Groups**

**1.      What is your role?**

a. Could you please describe this role?

**2. What involvement have you had in the WSICP?**

a**.** How have you been able to fulfil your specified role requirements?

**3.      What was your understanding of the WSICP?**

a.          What was your understanding of the WSICP before you became involved?

b.          How has this changed over time?

c. What training and education have you received?

**4.     Overall, do you feel that the investment of time and money in the ICP was worthwhile?**

1. Why is that?
2. What activities were of particular value?
3. Are there any you would advise against in the future?
4. Are there any other approaches to health service integration you would recommend for the future?

**5.  In what ways has health care become more integrated?**

a.          In what ways have you noticed WSICP facilitating a coordinated and shared approach to

patient care?

b. How has your capacity to refer patients changed through the WSICP?

c.     What barriers still prevent providers working more collaboratively?

d. What factors have been the most effective in supporting the delivery of integrated care in

Western Sydney?

e.      What are the main aspects that could be improved?

**6.**  **Let’s talk about different aspects of the WSICP and how they have impacted on your care of**

**patients and the integration of their health care. Could you please comment on..?** *(Explore*

*each point for experience and positive/negative aspects, gather examples, possible*

*improvements).*

a.          the patient-centred medical home

b.          the GP hotlines

c. Incentive/support payments for GPs

d.           Care Facilitator

e.          Use of Health Pathways Website for medical and service information

f.          Communication with other services, PHCP, allied health, community services etc.

f.1. Can you comment on the “quality” of that communication?

g.           IT systems

h.          Shared patient care plans

i.          The Rapid access hospital clinics

j.          Specialist action plan

**7. Concerning your relationships with other HCPs, can you please tell me:**

a. How your relationship with other HCPs has changed as part of WSICP?

b. How this has impacted on your working together?

**8.        Do you feel that you have been better able to manage the care of patients as a result of the**

**WSICP?**

a. Why do you feel that way?

b. In what ways has it changed management?

c. How have your skills in managing chronic patients developed/improved?

**9. What aspects of the WSICP have been the most important for you, for example, in managing your patients’ care? In integrating health care? In increasing your capacity to provide integrated health care?**

a.       Which have been less useful?

b. How much effort have you needed to put into these activities? What do you think is the cost benefit for you?

c. What has been the impact to “work flow” through your practice?

d.       What additional strategies would you recommend for improving the integrations of health care in western Sydney?

e.       What activities do you think should continue? Should cease?

f.       What would improve the WSICP in the future?

**9.       How do you think patients experienced the WSICP?**

a.          Which components of WSICP do you think helped patients to achieve better health

outcomes?

b.          What challenges/barriers do you think they have encountered?

b.1 Are there some patients who are not accessing the program?

b.1.1. Who are these (e.g. CALD, Aboriginal and Torres Strait Islander)?

c.           Can you give any patient examples which illustrate changes which occurred?

**10.       What impact did enrolment in the Integrated Care program have on:**

a.          The patient’s ability to navigate the health system

b.          The patient’s ability to manage their health?

**11.** How do you think the system has changed for the better as a result of the WSICP?

**WSICP interview introductions and conclusions**

**For Patients and Carers**

Thank you for taking the time to take part in this interview. The purpose of this interview is to gather your experiences of the western Sydney Integrated Care Program. The WSICP is where the hospital and GP services in Western Sydney have been working together to try and improve health care for people with chronic health problems by starting up some programs to improve the way they work together. We are aware you have been involved in some of these programs.

Feel comfortable sharing anything that comes to mind, including ideas, opinions, stories and examples. It is important to remember that your involvement is confidential and your participation is voluntary. This means that you don’t have to answer any question that you are uncomfortable with and if you wish to withdraw from the study, you can do so at any time without consequences up to the time we add the information from this interview to our overall data. (If a focus group, the following is added: “Whilst all care will be taken to maintain privacy and confidentiality, you may experience embarrassment if one of the group members were to repeat things said in a confidential group meeting”). Do you have any questions before we begin?

End of interview:

I believe we have covered all the questions I needed to ask. Before we finish, is there anything you feel I have missed or do you have anything you would like to add?

Thank you once again for your time and participation in this project.

Before we conclude here today, I would like to ask whether you would be interested to participate again at a later date within the next 12 months. *(If agreeable, a “consent to approach” form is completed with the participant to record preferred contact details).*

**WSICP interview introductions and conclusions**

**For Primary and Allied Health Care providers, Community Services, Hospital Services, Evaluation Advisory Group/Clinicians Working Group**

Thank you for taking the time to take part in this interview. The purpose of this interview is to gather your experiences of the western Sydney Integrated Care Program. Feel comfortable sharing anything that comes to mind, including ideas, opinions, stories and examples. It is important to remember that your involvement is confidential and your participation is voluntary. This means that you don’t have to answer any question that you are uncomfortable with and if you wish to withdraw from the study, you can do so at any time without consequences up to the time we add the information from this interview to our overall data. (If a focus group, the following is added: “Whilst all care will be taken to maintain privacy and confidentiality, you may experience embarrassment if one of the group members were to repeat things said in a confidential group meeting”). Do you have any questions before we begin?

End of interview

I believe we have covered all the questions I needed to ask. Before we finish, is there anything you feel I have missed or do you have anything you would like to add?

Thank you once again for your time and participation in this project.

Before we conclude here today, I would like to ask whether you would be interested to participate again at a later date within the next 12 months. *(If agreeable, a “consent to approach” form is completed with the participant to record preferred contact details).*


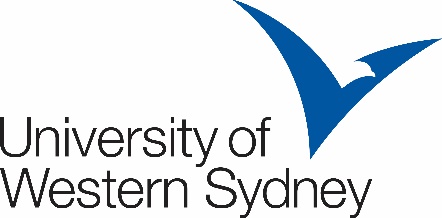
 [
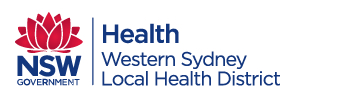
](http://wslhdold.staging.elcomcms.com/)

**Interview Schedules (WSICP Qualitative Evaluation) Round 2**

**Interview Questions for Patient/Carer**

**1.       What was it like taking part in the WSIC program?**

a.       What services did you have access to?

b.      In what way have your experiences of health care changed compared to your previous

experiences:

since before the program

since I spoke with you last

c. What can you say about support you received after joining the WSICP?

d.       Which components of the WSICP were most helpful to you - in improving your health?

e.      What do you think could be improved?

**2.       Could you please tell me about your experience of the following (if applicable)?** *(For each point explore how these helped or didn’t help):*

a.    Being allocated a Care Facilitator?

b. The Relationship Manager (or GPLN when role confirmed)

c. The rapid access hospital based service?

d.    A shared care plan? (The Care plan is provided by your GP and shared with the care team - patients access this electronically through Linked-EHR).

e. Specialist Action Plan? (The specialist Action Plan is provided by the hospital and contains recommended actions you can do to manage your health)

**3.       Thinking more about how your health care may have changed as a result of WSICP:**

a Who is the main person in charge of your care?

1. How well does this person understand you and your condition?

b. Were there any changes in the way your GP managed your care while taking part in the

WSICP?

                          i.      What were they? Can you give me an example?

                          ii.      Were there any changes in the way the general practice you attend was set up?

iii. When you went to see the GP were there any changes in the way your visit there

proceeded?

iv. Did you spend more time with other staff in the practice e.g. practice nurses than

you did before?

v. How did community health services assist in your health care?

c.      Do you feel that the GP and other practice staff communicated well with you and with

others outside the general practice (e.g. hospital and community HCPs)?

                           i.      Do you think the WSICP program led to any improvements in communication

between GPs and hospitals as regards your own health care? (E.g. through

shared care plan and rapid access service, in working as a team).

ii. What improvements have you seen in communication with community based

services for your own health care?

d.      Have you got any views on how this program may have assisted your GP? / Your hospital

nurses and doctors?

**4. How did your experience in the program affect your ability to:**

a.      Get the care you need from the health system?

b.      Manage your own health condition (e.g. having as much involvement as you like in decision

making?)

c. Were there times when you did not attend an appointment?

i. Please explain?

**5. What have you learned:**

a.      About yourself/your health?

b. About getting the care you need from the system

c. Are there better ways you could get the health care you need?

Please explain.

d. How has your understanding of Integrated health care changed since we last spoke?

**6.      What challenges do you still encounter or believe that you will continue to encounter?**

a.       Access

b.      Waiting times

c. Are there gaps between seeing the doctor, going for tests and getting results?

d. Cost

e. Transport and parking

f. Communication and spending sufficient time with you

**(WSICP Qualitative Evaluation)**

**Interview Questions for Service Providers and Advisory/Working Groups**

**1 What if anything has changed about your involvement in WSICP?**

a**. Since we last spoke h**ow have you been able to fulfil your specified role requirements?

**2      How has your understanding of WSICP changed since we last spoke?**

d. What training and education have you received?

**3.     Overall, do you feel that the investment of time and money in the ICP was worthwhile?**

1. Why is that?
2. What activities were of particular value?
3. Are there any you would advise against in the future?
4. Are there any other approaches to health service integration you would recommend for the future?

**4.  In what ways has health care become more integrated?**

a.          In what ways have you noticed WSICP facilitating a coordinated and shared approach to

patient care?

b. How has your capacity to refer patients changed through the WSICP?

c.     What barriers still prevent providers working more collaboratively?

d. What factors have been the most effective in supporting the delivery of integrated care in

Western Sydney?

e.      What are the main aspects that could be improved?

**5.**  **Let’s talk about different aspects of the WSICP and how they have impacted on your care of**

**patients and the integration of their health care. Could you please comment on..?** *(Explore*

*each point for experience and positive/negative aspects, gather examples, possible*

*improvements).*

a.          the patient-centred medical home –e.g. What has WSICP done to develop PCMH? Your role in PCMH? Value of PCMH? How does PCMH impact on WSICP

b.          the GP hotlines

c. Incentive/support payments for GPs

d.           Care Facilitator

e.          Use of Health Pathways Website for medical and service information

f.          Communication with other services, Private HCP, allied health, community services, Health ONE etc.

f.1. Can you comment on the “quality” of that communication?

g.           IT systems

h.          Shared patient care plans (Initiated by the GP) (Do hospital HPs and CFs add comments to this?)

i.          The Rapid access hospital clinics

j.          Specialist action plan (initiated by the hospital) GP awareness of these? How different from a discharge letter?

k. ***I would like to just talk a little about case conferencing.***

- Has this been offered to you? (Or if provider of c/c- “what is your involvement with this?”)
- By whom (e.g. the hospital and the discipline)
- Could you please describe your experience with this?
- What is (has been) the value of this?

**6. Concerning your relationships with other HCPs, can you please tell me:**

a. How your relationship with other HCPs has changed as part of WSICP?

b. How this has impacted on your working together?

**7.        Do you feel that you have been better able to manage the care of patients as a result of the**

**WSICP?**

a. Why do you feel that way?

b. In what ways has it changed management?

c. How have your skills in managing chronic patients developed/improved?

**8. What aspects of the WSICP have been the most important for you, for example, in managing your patients’ care? In integrating health care? In increasing your capacity to provide integrated health care?**

a.       Which have been less useful?

b. How much effort have you needed to put into these activities? What do you think is the cost benefit for you?

c. What has been the impact to “work flow” through your practice?

d.       What additional strategies would you recommend for improving the integrations of health care in western Sydney?

e.       What activities do you think should continue? Should cease?

f.       What would improve the WSICP in the future?

**9.       How do you think patients experienced the WSICP?**

a.          Which components of WSICP do you think helped patients to achieve better health

outcomes?

b.          What challenges/barriers do you think they have encountered?

b.1 Are there some patients who are not accessing the program?

b.1.1. Who are these (e.g. CALD, Aboriginal and Torres Strait Islander)?

b.2 Are there patients who do not attend follow up?

b.2.1. What might be some of the reasons behind this?

c.           Can you give any patient examples which illustrate changes which occurred?

**10.       What impact did enrolment in the Integrated Care program have on:**

a.          The patient’s ability to navigate the health system

b.          The patient’s ability to manage their health?

**11.**

a. What has changed since our first interview?

b. How do you think the system has changed for the better as a result of the WSICP?

**WSICP interview introductions and conclusions**

**For Patients and Carers**

Thank you for taking the time to take part in this interview. The purpose of this interview is to gather your experiences of the western Sydney Integrated Care Program. The WSICP is where the hospital and GP services in Western Sydney have been working together to try and improve health care for people with chronic health problems by starting up some programs to improve the way they work together. We are aware you have been involved in some of these programs.

Feel comfortable sharing anything that comes to mind, including ideas, opinions, stories and examples. It is important to remember that your involvement is confidential and your participation is voluntary. This means that you don’t have to answer any question that you are uncomfortable with and if you wish to withdraw from the study, you can do so at any time without consequences up to the time we add the information from this interview to our overall data. (If a focus group, the following is added: “Whilst all care will be taken to maintain privacy and confidentiality, you may experience embarrassment if one of the group members were to repeat things said in a confidential group meeting”). Do you have any questions before we begin?

End of interview:

I believe we have covered all the questions I needed to ask. Before we finish, is there anything you feel I have missed or do you have anything you would like to add?

Thank you once again for your time and participation in this project.

Before we conclude here today, I would like to ask whether you would be interested to participate again at a later date within the next 12 months. *(If agreeable, a “consent to approach” form is completed with the participant to record preferred contact details).*

**WSICP interview introductions and conclusions**

**For Primary and Allied Health Care providers, Community Services, Hospital Services, Evaluation Advisory Group/Clinicians Working Group**

Thank you for taking the time to take part in this interview. The purpose of this interview is to gather your experiences of the western Sydney Integrated Care Program. Feel comfortable sharing anything that comes to mind, including ideas, opinions, stories and examples. It is important to remember that your involvement is confidential and your participation is voluntary. This means that you don’t have to answer any question that you are uncomfortable with and if you wish to withdraw from the study, you can do so at any time without consequences up to the time we add the information from this interview to our overall data. (If a focus group, the following is added: “Whilst all care will be taken to maintain privacy and confidentiality, you may experience embarrassment if one of the group members were to repeat things said in a confidential group meeting”). Do you have any questions before we begin?

End of interview

I believe we have covered all the questions I needed to ask. Before we finish, is there anything you feel I have missed or do you have anything you would like to add?

Thank you once again for your time and participation in this project.

Before we conclude here today, I would like to ask whether you would be interested to participate again at a later date within the next 12 months. *(If agreeable, a “consent to approach” form is completed with the participant to record preferred contact details).*
