# Supplementary material for: Integrating health care in Australia: a qualitative evaluation
Source: BMC Health Serv Res. 2019 Dec 11;19:954. doi: 10.1186/s12913-019-4780-z (PMC6907151; doi:10.1186/s12913-019-4780-z)
Supplement: Supplementary file 3 — Additional file 3. Matrix coding of specific WSICP initiatives with illustrative quotations. [file 12913_2019_4780_MOESM3_ESM.docx]

**Additional file 3.**

**Matrix coding of specific WSICP initiatives with illustrative quotations**

The following analysis of the interview data is organised in a matrix specifically addressing each of the key WSICP strategies: Care Facilitators, IT Systems, Shared Care Plans, Specialist Action Plans, GP Support-line, Rapid Access and Stabilisation Service Clinics including Patient Hotline, HealthPathways Website, Support Payments for GPs, Patient Centred Medical Home development, and communication with Non-WSICP services.

As agreed in our research plan, each of these strategies is described according to implementation/delivery, use, experience (positive/negative), satisfaction, perceived value, and suggestions. Implementation/delivery is process oriented and considered as “Developing, operationalising and implementing strategies”. Use is conceptualised as “How patient, carers and health care providers make use of the strategy”. Experience (positive or negative) is regarded as “What happens to the participant.” Satisfaction is the “Affective response to experience”. Perceived value is regarded as “Judged usefulness to self and others”.

**Participant Key:**

MG (Management Group); HS (Hospital Specialist); HN (Hospital Nurse); AH (Allied Health); GP (General Practitioner); PN (Practice Nurse); CF (Care Facilitator); PC (Patient/Carer).

*Black font indicates first round interview (noted as Round1)

*Blue font indicates second interview of first round participant (noted as Round 2).

*Purple font indicates new second round participant (noted as Round 2).

| **Care facilitator** | |
| --- | --- |
| **Implementation / delivery**   - *Employment by LHD with role in the community sector may be challenging but is being achieved* - *Unclear roles but developing with program* - *Uncertainty of future CF role* - *No guidelines or procedures for CF* - *Inconsistent instructions* | * you could look at the placement of the coordinators and the role of the coordinators, whether they should be rather more firmly based in the community health services, maybe in the PHN, practice based, rather than being very clearly employed by the LHD, what difference that makes to their attitude, loyalties and behaviours. (MG3 Round 1)  *I think the care facilitators really are these days more community based than hospital based (MG3 Round 2)  * it is a fairly new role as well and we’re pretty much the guinea pigs at the moment. (CF3 Round 1)  *we need to clarify what their role is in regards to how many patients are their patient load or whether they do need to do more heavy duty end and some of the monitoring end is done differently. So we're really starting to model around that (MG6 Round 2)  * The care facilitators who are integrally involved in helping us with this hadn’t really worked out their roles either. So I think that’s taken a while for each group in this to work out what your role is (GP5 Round 1)  *care facilitators are kind of stuck in the middle, one is never quite sure whether they’re there because they need to be a permanent and ongoing feature in the system or whether they’re there as a compensatory mechanism until we can end up with good care coordination in the community working between general practice and community health (MG3 Round 2)  * we were just sent out as crash test dummies to try and figure out what was the best way to engage GPs and patients into the program. (CF1 Round 1)  * we were never given a guideline, we were never given a procedure, we were never given a policy. We were just given word of mouth of what they wanted from us and it differed. And because it was a partnership WentWest saw one version compared to the LHD. (CF1 Round 1) |
| **Use**   - *follow up post discharge and notify hospital* - *proactively arrange hospital appointments and reminds patients* - *Others providing CF role? duplication* - *Providing contact with GP* - *Provides useful information for HCPs and PCs and advocacy* - *Patient education* - *Develop patient care plan* - *Link between HCPs* - *More engagement with CFs* | * …they follow up with the patients we see when we discharge them, and they let us know what's happening with them and remind patients to come and see us, so they're good value. Yeah, we work quite closely with care facilitators…(HN4 Round 1)  * she got me onto the endocrinologist, because I had diabetes for 16 years and at the beginning, I learnt all about it, and I saw endocrinologists, and just sort of took the tablets, and lost a bit of weight (PC3 Round 1)  * She just rang the other week and then I actually forgot about - to get back to her, and then she just rang again and said, “Tomorrow you're going to the clinic, I'll see you there." I said, “Yeah, sweet.” (PC4 Round 1)  * I think [CF] is an additional help for me and also for the patients as a reminder system to them. (PN6 Round 2)  it took a long while because in some ways we’re still trying to find our roles, for example, well, who does the follow-up on the patients? (AH4 Round 1)  *… I was looking to contact them [CF] for them to try and coordinate these appointments, but I just thought it was easier for me to directly contact the endocrinologist and say, look, we already have a clinic which is running tandem, would you mind seeing them? (AH5 Round 1)  * … if I’m actually having problems with contacting the GP for getting information with regards to the patients … I usually refer it to the care facilitators. They’re actually great at giving me the information required. (HN6 Round 1)  * …really good in communicating with us. We've had a couple of patients admitted to hospital that we didn’t know of… it just kind of helped us to try and get in contact with them and make sure that we follow up with them after that acute episode (PN4 Round 2)  * a lot of GPs tell me that, there’s a big gap in terms of when patients are being discharged from hospital and they don’t get an appropriate discharge summary or they’re given to the wrong patient, or they’re not even aware that their patients have gone into hospital before our service came on board. (CF3 Round 2)  * … I'll often have questions and they may not be related to the integrated care but at least she might say to me no you need to go here, there or whatever. So I'm using her as a resource for everything, actually. (PN2 Round 1 )  * If I have a question – I just quickly jump on the phone with [CF] and she’ll say, “Yep, no worries, I’ll email the doctor,” And if I have to get a referral to someone I wanted to check with [CF] if there’s any information about it. She goes, “Don’t worry, I’ll send it. I’ll send it off to the doctor. Don’t worry, I’ll get it sorted,” and it’s done(PC1 Round 1)  *I think I’ve only got to make a phone call and they’re just onto what I need.(PC8 Round 1)  *I think if I couldn’t get over there – which is difficult to get over there anyway – I probably ring up [CF], ‘cause I don’t want to bother [HN] every five minutes, so [CF] is the one that comes to help me. (PC11 Round 1)  * Often some of the patients don’t know what services they can get or what help they can get. Sometimes just having an advocate, having an educator or someone with them, has a big impact on definitely service navigation and the understanding of their chronic disease. (CF3 Round 2)  *I was ringing her a lot, I was really frustrated at different times and I rang her and said what do I do [CF], you know, I’m after this, I’m after that, keep pushing it, ask for it (PC19 Round 2)  * the previous care facilitator was trying to help them quit smoking and discussed options and different management techniques … They have been very helpful… (GP4 Round 2)  * Care facilitators, like when we see people in clinic we contact them and they go out and talk to the GP how to best manage, come up with a plan and tell them about the services and then work with them so that the patient can be well-managed. (AH1 Round 1)  *I’m looking at the care plan that she has done they’re very detailed. She has been talking to me about how she sets up – there’s some patients she reviews monthly, some patients she reviews every two weeks, so she’s – keeping up with – she seems to know what she’s doing (PN7 Round 2)  * the care facilitator making that physical dynamic real time link between the care providers.(MG5 Round 1)  * … and whenever we try and link up a new patient with the care program they also try, if appropriate, try and meet the patient at the clinic (GP4 Round 2)  *she's actually linked them in with other services that I haven't been able to link into or haven't heard about (PN4 Round 2)  *… much, much more involvement now with the care facilitators than there was beforehand. (HN5 Round 2) |
| **Experience (positive**)   - *Educating patients* - *Educating HCPs* - *Coordinating care* - *Understanding HealthPathways* - *Maintains contact with patients and carers* - *Organises appointments* | * today, I had a patient who’s a newly diagnosed CCF and [CF] is brilliant, I was sitting here listening to her educate the patient on his  newly diagnosed disease and learning from her, she really is very, very good. And the family were really pleased and I could sense  that they felt quite comfortable with her knowing that she would also be a part of the team if they were in hospital or sent down to  the RASS clinic and I think it was very well done. (HS9 Round 1)  * She helps me a lot and I’ve learnt lots from her… (PN1 Round 1)  *I’ve had some good experience with the care facilitators. We’ve had a case conference this year with a patient who required a lot of  input and a lot of work between various groups, and she co-ordinated everything; she was fantastic. (HN2 Round 2)  * The care facilitator is a living breathing HealthPathways. They know the things in behind the walls so they can match the  requirements up really well. So I think from that point of view, totally brilliant. (MG5 Round 1)  * She would regularly keep – initially we had the one-on-one meeting with Mum present and then she would regularly be in touch  with whatever, just follow up any issues, anything that I can help you with, you know, which I thought was fantastic (PC17 Round 2)  * she has helped me so much. She’s got me letters from the diabetic place and she sends me every little pamphlet she finds, and she  rings me up to see how I’m going all the time. (PC10 Round 1)  * I speak to [CF] maybe once a month; she’ll ring and check in, which is awesome. (PC1 Round 1)  * in the hands of the care facilitators … seeing more services and more timely services for their patients for the care plans. MG5 Round 1) |
| **Experience (negative)**   - *Infrequent contact from CF* - *CF is “face” of the program for GPs and experiences their frustration* - *CF unsupported* - *Role of CF not respected/valued* | *I guess it’s nearly six months since I first made contact with [CF]. I guess there was a period there where there was – I hadn’t heard from her for a while. Whether she was busy or on leave, or had too many patients, I don’t know. .(PC3 Round 1)  * being the care facilitator, you’re the face of the program in general practice, so if …anything’s not working you’re pretty much the one that cops it from general practice, ‘cause they say, ‘oh, we were told this was going to happen and that’s going to happen’, so they obviously tend to disengage with you as well if the IT systems are not working or if you’re taking too much of their time on a system that’s not functioning … (CF3 Round 1)  * never had any backing. When we come across a problem we used to have to self-solve our issues …no backing. (CF1 Round 1)  *We've got a GP that just thinks I'm some admin officer, doesn't want any communicating from the care facilitator at all.(CF2 Round 1)  *one GP that I tended to contact, and he is just dismissive, and there’s another GP on the program that doesn’t want any input from the care facilitators either, just admission and discharge notification (CF2 Round 2)  *Before we used to be a nurse – they [GP] used to think we’re just the nurses, but now I notice that’s changed and they feel the importance, of having us in there and trying to help them to navigate their patient for proper care and management. (CF4 Round 2) |
| **Satisfaction**   - *Professional and good at the job* - *HCPs learning from CF who provides information* - *Reminds and guides patients* - *Provides contact for hospital with GP* - *Input to care planning and coordinating appointments valued* - *Close relationship and contact with HCPs and patients* | * Well, she was fairly magic for me…, and she was certainly interested in my progress, and I think she wasn’t aware of everything that had happened to me, and it was always just very professional. (PC9 Round 1)  * I think they're great at their job. Yeah, yeah, they're good (HN4 Round 1)  * …she’s good at her job and she knows her stuff and I have learnt (PN3 Round 1)  * She's great. She's fantastic. I mean I constantly ring her and ask her and she guides me; she's fantastic. (PN2 Round 1)  * Yeah and remind me, yeah, because sometimes - I'm not good with memory and I mixed up the days. Very good. (PC5 Round 1)  *She’s very good. Anything that I want, she says to give her a ring, and she makes appointments for me(PC11 Round 1)  * … coming to the practice, so if we have got a patient we want her to get her to follow-up with, or she has been telling us who she has followed up with …that's been really good for structuring for planning the care as well (GP6 Round 1)  *our care facilitator, he’s excellent, and we’ve been told about his care plans, he’s then documenting in the patient’s notes, they’re great, he’ll ring me and tell me, “I’ve seen so and so,” and he writes in the notes, and that’s actually quite useful (PN2 Round 2)  *It's very good… if I have problems with a patient, especially with organising their appointments, they actually help me with regards to facilitating that (HN6 Round 2)  * …without her input I dare say I’d be struggling. And she has her assistant, she phones you every second week to see how you’re going. Are you doing this and what’s happening with you. (PC2 Round 1)  *She is always checking in with us-how things are going, anything else we need-we know she’s there if we need her (PC15 Round 2)  * …there’s one care facilitator, she’s fantastic. She’s a very close relationship. We actually email each other all the time, we talk about patients. When a patient comes and she’s available to be here, she will be here to support the patient. And she’ll refer. She has great… working relationships with GPs. So that’s fantastic (HS7 Round 1)  *We had one, she was great, she’s left, and I have a new one that I haven’t actually made much contact with. And, certainly when we had , she was very helpful (GP14 Round 2)  *There are some really good care facilitators. They're amazing. The patients like them, patients know them, they know patients really well. They communicate with me with any concern the patients have or anything that's important for us to be aware of. (HS7 Round 2) |
| **Perceived value**   - *Follow up of patients post discharge and communication with hospital valued* - *CFs are a big improvement in care delivery* - *Consistency and familiarity important for patient/ carers* - *Holistic focus of CFs* - *Face to face contact useful* - *Patient assessment and input to care planning* - *High expertise of CFs* - *Assists HCPs in navigation through health and other services* - *Linking hospitals and community based services* - *Links hospital, GP and patient* - *Assist in clinical follow up of patients and provides information* | * they're a good link because they follow up with the patients we see when we discharge them, and they let us know what's happening with them and remind patients to come and see us, so they're good value. Yeah, we work quite closely with care facilitators and keep in touch. (HN4 Round 1)  *I’ve got faxes - they turned up in emergency or in the various hospitals. And that part has been extremely helpful. (GP9 Round 2)  * The care facilitators are superb, they, I think, are the lynchpins of it all (AH4 Round 1)  * the care facilitator, that's been probably the biggest person that has become involved in our patient care that wasn't previously involved in the care, so yeah, so having that person on board has been a big improvement, definitely (GP6 Round 2)  *the care facilitator is able to come out to the community and meet the patients; I think that's quite good. I think the role of the care facilitator is actually quite vital and important (GP4 Round 2)  * it is very helpful because I think the patient does feel that they have consistency and that makes people feel comfortable when they see a face and they know they’ll see that face somewhere else (PN3 Round 1)  * having the care facilitator or a support person or a go-to person is what they value the most because they know that person is consistent and was up-to-date with their care, and they can talk to us pretty much about any even social issues, mental health issues, emotional wellbeing, holistic kind of care that we provide (CF3 Round 2)  * I think having the care coordinator look at the patient’s care plan and also them having done an assessment of the patient and that assessment being communicated to the GP, that has been particularly valuable. So we've picked up extra things, extra issues that we weren't aware of. The other thing is just having someone to make sure that the patient is navigated through the health system. Someone the patient can call if there's an issue; that’s been very valuable for the patients.(GP1 Round 1)  * I’m really, really, really confident and encouraged in terms of the work that the care facilitators do and how – they’re just competent, they’re a high calibre of nurse that we have. (MG2 Round 1)  *care facilitators are kind of stuck in the middle, one is never quite sure whether they’re there because they need to be a permanent and ongoing feature in the system or whether they’re there as a compensatory mechanism until we can end up with good care coordination in the community working between general practice and community health (MG3 Round 2)  * having that care co-ordinator has been the most useful thing for me so far, so I’m just getting an idea of what services there are… all of sorts of things from taxi vouchers to payments for incontinence pads, all that sort of stuff (GP2 Round 1)  *that central care facilitator who knows what is going on and that they know how the hospital works and they know what's available and they know where the services are and things (GP6 Round 2)  * Definitely having the care coordinator, that has got to be maintained, that's really important having that person here as the link between the hospital and you and the patient, and who has got to oversee everything, that's really vital (GP6 Round 1)  * I think having the care facilitators work in directly with the private care practices and the GPs is helping build stronger links between the hospital services and community services and primary care. (MG6 Round 1)  * Oh, [CFs] really good, because sometimes I get a bit vague about making some of these appointments …I’m not getting a letter from them so I get on to [CF]. And [CF] is pretty good. (PC2 Round 1)  * Yeah, my sugar was really high; really, really high, yeah. Now I’m trying to get it down and [CF] is ringing me up all the time checking up, which – you know what? It does help. It does help. (PC10 Round 1)  * That’s good. She rings me every now and again and asks me what’s happening and everything else, and if I’ve got a problem or that, she intervenes. (PC12 Round 1)  *Very good. Yeah. They’ve told me things that I’ve had no idea that existed. (PC12 Round 1) |
| **Suggestions**   - *Maintain the CF role* - *Flexibility in CF role* - *Clear policies and procedures* - *Ensure practices are prepared before introducing CF* - *More home care* - *CFs could provide greater assistance with community based care options* - *Review CF workload* | * Definitely having the care coordinator, that has got to be maintained, that's really important having that person here as the link between the hospital and you and the patient, and who has got to oversee everything, that's really vital (GP6 Round 1)  * the care facilitator role needs to be more flexible than we’ve designed it because the practices, the general practices are different across the whole spectrum and so each scale and size and organisational level of practice dictates a different need of the care facilitator. (MG5 Round 1)  * There's no policy, in procedures in place, so you're really opening up nurses to a lot of litigation should something come - go wrong in the field. So those things need to be addressed (CF2 Round 1)  * … doing a lot more work in the primary care GP practice …so that they’re integrated care ready …Once they’re ready and they’ve had a lot of support then we bring the care facilitator in. We’re kind of taking the care facilitator in too early…So now what we’re thinking is maybe splitting that role so that you’ve got the back end function supporting the GP and then once they’re ready then bringing in the front end function which is about care coordination and patient focus stuff. (MG6 Round 1)  *Community care and that, they keep you in your own place. I think there should be more of that out there so that people know. I think it should be, I don’t know, but I had no idea that you could stay in your own place and they would look after you.(PC12 Round 1)  *if they're [CF] coming out to do a home visit or something, that would be something they could be checking, do they have a computer, do they have an iPad, how are they accessing their health information - have they ever accessed it, that kind of thing (GP6 Round 2)  *I’ve just recently done a HealthPathways on the NDIS, so I feel that that’s something that the care facilitator should be really up on, and community transport options. I had a COPD patient who I had to work out all this myself – complicated. Maybe if they had a checklist of - discuss transport options and give transport options if these are trouble for a patient. (GP5 Round 2)  *there needs to be some work around what is really an appropriate number of patients and how many hours up to – 200 or something patients already, you feel you’re drowning, because you’re just going out to meet with patients at practices, you’re doing assessments, you’re doing telephone reviews, then phoning the GPs about what hospital to discharge, you’re doing referrals and needing to make sure the GP stays on board and engaged you’re sending faxes every day, writing, all this takes time and when you have a huge amount of patient numbers, you’re not really able to provide the proper care that these patients need. (CF3 Round 2)  *if they're [CF] coming out to do a home visit or something, that would be something they could be checking, do they have a computer, do they have an iPad, how are they accessing their health information - have they ever accessed it, that kind of thing (GP6 Round 2) |

| **IT systems** | |
| --- | --- |
| **Implementation / delivery**   - *Hospital to GP communications was not prioritised at inception* - *Poor web presence* - *Poor capacity for data collection and QI* - *System unable to accept clinical metrics*   ***GP Issues***   - *GP time required* - *Under resourced* - *Flawed IT threatens GP engagement* - *Delayed delivery* - *Challenges with GP software including multiple systems* - *End user training required*   ***Hospital issues***   - *Outdated systems* - *Different templates within the hospital* - *Hospital doctors not aware or accessing information uploaded by GP* - *Time expenditure in using*   ***Connectability Issues***   - *Different systems – incompatibility also duplicates work* - *Difficulty uploading care plans* - *Challenges linking between hospital and community* - *Delays in implementation of connectable systems* - *No e-health* - *Possibility of linking with private specialists* | *I would have thought that the step of communicating with the practices to have been worked out from the beginning. It seems like it’s not and that surprised me a little bit because that’s the whole point of all this, is to share the information (PN7 Round 2)  *the most frustrating and complex area of the whole demonstrator. It would probably be one of our biggest learnings. Some of the key functionality that was meant to be in phase one…our existing E-referral, that’s still not up and functioning (MG6 Round 2)  *at the beginning there was a lot of resources devoted towards the IT aspects of integrated care, but I just feel like it takes a long time to make any change. (HS3 Round 2)  * I’m not convinced that we have good just general web information presence on our information systems…Patients still can’t find clinics… these days everybody looks to the web and the internet to find information. (HS1/MG7 Round 1)  * Data collection is crucial for this program to work and to demonstrate that it’s working and I think the data collection and input into the program has been quite flawed (HS11 Round 1)  *we were trying to establish putting everything electronically to be able to easily pull data but we’re not able to do that. So, we’re trying to keep our own audit… (HS3 Round 2)  *clinical metrics can’t be uploaded into Linked-EHR (CF2 Round 2)  *they’re spending a lot of time on it …spending with the people from WentWest…and then they’re onto PenCat …they can’t keep up. (CF1 Round 1)  *In terms of the kind of investment that goes into developing IT systems more broadly, it’s very, very tiny, and the Linked-EHR suffers from lack of investment generally (MG3 Round 2)  *… the doctors don't understand what it is either. It’s worse when Top Bar is not working ...like they'll say, “Well what is this integrated care?”(PN2 Round 1)  *I'm assuming our system is linked up electronically, I don’t know. We’re just not very IT wise here (PN2 Round 2)  *… I went to develop care plans and the error message came up. So there was one message and then we got through and then we tried to develop another one and then we got so far into the care planning and a different error message… very reliant on the way they code their Best Practice and Medical Director and if the identifier is not correct from Medicare you’ll come up with an error.(CF1 Round 1)  *Oh, my gosh. From then until now, oh my goodness. The top bar was up and down. And recently, it was down (PN1 Round 2)  * …IT systems are so fragile that maybe they can’t cope with another software with Linked EHR (CF3 Round 2)  * It’s just that it’s not an easy set up. It requires a physical engagement, enrolment, usually taking WentWest’s staff to do it. It requires some degree of training of the end user.(MG5 Round 1)  *…CERNER it’s a bit outdated…it’s never actually tried to be updated to be a bit more functional on the GP side (HS5 Round 1)  * … we're being forced to use Cerner because that's the one that New South Wales Health and others have adopted. What I use for case conferencing… is Genie, which is just like the GP software. HS8/MG8 Round 1)  *…within our hospital systems, like all the clinics work really differently. Like heart failure clinic is so different to diabetes clinic or COPD clinic. The letters that go out to GPs, the templates are also different. (CF3 Round 1)  *OTs or the physios that may have been in with the patient, the ability to be able to actually access their notes on line because at the moment OTs and physios try and use electronic records and that’s a big issue (AH4 Round 2)  *I had uploaded a lot of information about the patient onto their eHealth record or eHealth system but I don’t think the hospital doctors were aware of that. I don’t even know if they had access to the care plans as such…, so I think there’s still problems with accessing the same information about a patient(GP2 Round 1)  *We can’t access the shared care plans – through the server we can actually link in with the EHR (HN4 Round 2)  * A few of the doctors are able to do it [access EHR] and they’ve been doing it. If they don’t find anything on it they usually ask us anyway. But apart from that there are always doctors there who don’t know how to access it to be honest (CF4 Round 2)  * after seeing a patient, we have to write, put in data, put in a report and it takes a lot of time to actually enter the data into it and they would have to generate another specialist letter to the GP. All that takes time. (HS7 Round 1)  * some of the allied health don't have the same computer programs if we’re having to fax instead of being able to shoot things off. (PN9 Round 1)  *Well the letter that we write at the end of consultation is still posted - we rely a bit on snail mail (HS4 Round 2)  *I’m ringing a GPs rooms to say, “Can you please send me the current medications,” there are some GP practices that you actually have to write a letter requesting it formally (HN5 Round 2)  *it’s [Linked EHR] still not compatible with all the systems. I think it’s only compatible with best practice and medical director software (GP5 Round 2)  *… we use the care plan per patient and Best Practice software that we use. And the integrated care program is using a different template so they are basically creating two different care plans (GP13 Round 2)  *I think at the start of this, we really thought that integrating the e-health systems would happen. But one of the biggest frustrations is that that has not happened, and I suppose though, maybe it’s because it was a more complex beast than we potentially (HS1/MG7 Round 1)  *like most of them [GPs] are not even able to upload shared care plans because the system’s not even working yet. So the hospital can’t see it. (CF3 Round 1)  *The connections between the GP and here are still not manageable because the electronic component isn’t up and running (HN4 Round 2)  * …different GP practices have got different computer programs, they don’t marry up with the computer programs that we’ve got, so we can’t see their information, they can’t see our information...(HN2 Round 1)  *It can still happen in what we do here and the technology and techniques that we’ve got, certainly the shared care plan, that technology’s available for viewing in private places, in private specialists MG5 Round 1) |
| **Use**   - *Duplication of data entry* - *Unclear whether other clinicians reading transmitted data* - *New referral forms built* - *Uncertainty regarding documentation in hospital database* - *Encrypted communication required for referrals* - *Time required for use of Linked EHR* - *Some GPs prefer non-electronic communication* - *CFs entering EHR data into hospital systems* - *Some use of electronic discharge letters* - *Fax still preferred method of communication* | * we are entering the data in our clinics and our letters, and then I’m having to use that information and then manually take that across into an Excel sheet, but there should be a way to automate that process. We shouldn’t have to doubly enter data.(HS6 Round 1)  *We do a lot of doubling up in terms of notes and that sort of thing. So we’ll have paper files, the research paper files, we have the hospital electronic notes. There is a lot of overlap (AH5 Round 2)  *… I’m just sending it out into the ether… just hoping that the person on the other side has actually read it and actually acknowledged it.(HS5 Round 1)  * We’re able to send and upload new care plans and that part of it’s working well. What’s happening with information coming back is what we need to work out. Not sure if we are getting it always (PN7 Round 2)  * so one of the doctors at Westmead has built the forms for – a dietician form and an educator form and an endocrinologist form, so we’ve got – I don’t write notes anymore, I type it all into the computer.(AH2 Round 1)  *All I know at the moment regarding IT is that, purely the documentation services, that there is something there on Power-Chart …a particular integrated care section to use.(HS10 Round 1)  *we’re documenting now in Cerner what’s going on, and Cerner is now extracting that and creating the letter which we modify, but the letter then gets posted; it doesn’t get sent via Linked-EHR or anything else, as far as I know (HS8/MG8 Round 2)  *It’s improving as more people have yet to take on encrypted communication… I just send my care plan electronically with the paperwork through the podiatrist. He will electronically send me back a report (GP7 Round 1)  *I do have access to Linked-EHR, and I have been shown how to use it. But I know some GPs have chosen not to use Linked-EHR because of IT issues. (HS7 Round 2)  *At this stage we’re putting notes into Cerner, so every time they access a document patient in the Cerner, if they see there’s a previous Linked-EHR updated, that’s how we’ve been doing it. (CF4 Round 2)  *Occasionally, I get an electronic discharge if it's been uploaded onto a patient's electronic, like, My Health Record. Then we'll be notified, but often that's not a routine thing. It seems to be a routine thing in the children's hospital actually, but not at adult hospital. (GP2 Round 2)  *there are some GPs and some cardiologists that have actually asked for email but only when their fax line is down, they still want the faxing (HN4 Round 2) |
| **Experience (positive**) | * |
| **Experience (negative)**   - *Database unable to produce required information* - *No audit facility for Database* - *Clinician needs not accommodated* - *Delays in responding to requests persist* | * it’s hard to not be frustrated with IT systems. We thought we’d go completely paperless with our – just our basic clinic records and we tried to do that, so we worked with IT initially we wanted to use more a database form, but they advised us to use Power-note because it would then print out into a letter that the patient could take it home… we use it, it doesn’t print out a proper letter. It’s a letter that gets spread out across multiple pages. It’s horrendous. (HS1/MG7 Round 1)  *My other sense is that the IT folk don’t understand what the clinicians want, and when we try to say to them we don’t want that, we want this, they don’t listen. (HS2/MG9 Round 1)  *the ability to audit the data; we still haven’t been – we’ve logged multiple jobs, and this has been over a six to eight month period and none of those have come back. So, unable to audit our own data, unable to write our letters as planned. (HS1/MG7 Round 1)  *Ohhh Westmead IT is just horrendous. It’s just getting anything done, we’ve got a job still in from July last year, we just keep following up on it. Yeah, yeah, we’ll get to it, we’ll get to it. July last year, really? (HN2 Round 1)  *just frustration because it was paid to work and it doesn’t and its coming and its coming and it’s still happening (HN4 Round 2) |
| **Satisfaction**   - *Letter formatting improved* - *Unable to receive e-referrals* - *Electronic notes vs hand written notes* - *Practice staff prefer Linked EHR* - *Improved IT assists prompt communication and follow up care* | * the care plan at the bottom of the letter, like the letter and the format I think is better than what I was sending them before, so that has certainly improved that sort of integration (HN4 Round 1)  * it’s taken a lot of work out of my day, because I no longer have to come back from the clinic and enter that data again, to the database. It’s also taken some work of out of the administrative staff, because they don’t have to copy, paste, and reformat a letter from Cerner (HS6 Round 2)  * the other frustration is that they said we could get e-referrals. We haven’t got any e-referrals from any of the external practices. I mean, we think that GPs should be able to e-refer; they still can’t e-refer to us at all.(HS1/MG7 Round 1)  *so one of the doctors at Westmead has built the forms for – a dietician form and an educator form and an endocrinologist form, so … I don’t write notes anymore, I type it all into the computer. So I feel like that’s been a huge enabling factor; it’s made me work quicker… (AH2 Round 1)  *So we use PowerChart and we write all of entries electronically, which can be turned into a letterhead. It is good in that … instant accessibility is important and if it’s online you can reproduce it, print it. (HS4 Round 1) *when it works it’s so much better than the medical software. I would much prefer to use Linked EHR all the time. (PN3 Round 2)  *I'm seeing changes or I'm learning about patients being in hospital much sooner. And therefore I'm able to follow up those patients a lot sooner as well. So within a day or two of them being discharged I'm getting to see them now. (GP9 Round 2)  *In the past, I mean patients, weeks after they’ve been discharged, missed the time that they wanted repeat tests done. Or the patient might even forget to bring their letter with them. And sometimes they won’t even tell you they've been in hospital. (GP9 Round 2) |
| **Perceived value**   - *Hospitals sharing care plans across disciplines* - *Too much effort required from GPs (not worth it)* - *On line documentation good* - *Good when fixed* - *Top Bar helpful in checking for care gaps* - *Patient access to online portal* - *When information can be shared efficiently and securely* | *… power chart…there is a separate section and that auto populates their plans into our care. So, if the patient is being seen by them [endocrine or respiratory], I’ll automatically know what their plan is from their perspective. (HS6 Round 1)  *technology has been more of a challenge rather than making life easier ‘cause it’s not working at the hospital end yet and it’s not working at the majority of the GP practices as well, so I’m not sure if the time and money we are spending in terms of linked EHR is really worth it because the majority of the feedback that I get from GPs, after two attempts they’re like, “I don’t want to do this. I don’t even know if it’s worth it.”(CF3 Round 1)  *… online documentation has been pretty good…typing straight into notes…the way of the future rather than having a big set of medical notes; just for everything to be online and integrated care is a bit of a pioneer leading the way. (AH2 Round 1)  * I feel once that’s fixed it’s probably going to be a good idea, and every time we see a patient we update their plans and our recommendations (AH2 Round 1)  *I know a lot of money has been put into PenCAT, which is great. I think it has a lot of potential but I know it takes a while. So, they’re still working on that. (GP5 Round 2)  * the portal is quite easy to use, the Top Bar is quite useful in terms of how it's sort of drawing out data from your Medical Director or the Best Practice Program to make sure you have got no care gaps - that's really good. (GP6 Round 1)  *Patients can have access to the online portal, which is really good, so they can see what has been changed or what their medication list is…They are also able to then access the clinics, the heart failure clinic… (GP6 Round 1)  *there is the ability to send the information directly between primary care doctors and the hospital in a timely way. Like, for the clinics, for example, having easy access to the clinics and then having the ability to upload information about the patient quickly.(GP2 Round 1)  *it's still very basic at this stage. I think it's still - the connectivity, I'm not really happy with. I mean, ideally everything should be live and whenever we make a change, it should change instantaneously. (GP12 Round 2)  *I think they’re still concerned about security with email. (HN2 Round 2) |
| **Suggestions**   - *Consider effective systems already available and in use* - *Strong decision making needed to deliver appropriate IT* - *“Compulsory” patient controlled e-health* - *Single system needed* - *Training needed and user friendly software* - *Change or change use of Cerner* - *Develop systems “before” program implementation* - *Start with paper based system and translate to IT based* - *Use of encrypted email* - *General practices flagged when changes made to patient records* - *Improved data collection for evaluation* | *we can do that with Genie and it does everything that Cerner is meant to do. Cerner is still getting there …It's clunky, it's clumsy. The way it's organised, it's not organised around patients (HS8/MG8 Round 1)  *I don't know how much involvement the specialist has in the Linked-EHR, but their ability to access the shared care plan would be helpful (PN4 Round 2)  *somebody has to make some strong and dictator like – some strong decisions to get us doing that. I mean, with non-compulsory patient control e-health records it doesn’t work. We even need to be – everyone on the same system or we can’t manage billions of different systems. It just doesn’t work. (HS1/MG7 Round 1)  *The thing that would make a really big difference would be if we could look at their notes, and they could look at, maybe not everything, but if I could actually look and see what's happened. If we had a shared electronic record. (GP7 Round 2)  *If the hospital was doing something they'd just send me a discharge letter and then I have to manually change the medications. In an ideal world, we would have a system where all the software programs can connect up and everything is live (GP12 Round 2)  *so we’re now working on E-referrals and sending out the information through the IT systems, so we could do with some more training from that side of things (HS5 Round 1)  *improving the IT a little bit more just to make it a bit easier to make the online care plan and things linked EHR and just getting at least as easy as possible, you know, as user friendly as possible; not everybody is as computer savvy as others (GP6 Round 2)  * working on the software, to make it as easy to use as possible and having a way for the hospital to also access that would be really use useful (GP2 Round 2)  *Or find a system that would interface with Cerner so that Cerner doesn’t have to do it all. It can become the repository of it but you have intermediary systems that are much more flexible and built for purpose and let the programs talk to each other. Don't force the providers to use the clunky Cerner thing… (HS8/MG8 Round 1)  *if we were starting again we should just develop our systems on paper, and this is with the experience in Christchurch as well – develop the systems on paper, make the systems work on paper and then get the IT to enable it, don’t do the IT first.(HS2/MG9 Round 1)  * … have everyone on an encrypted email and not have to be scanning thousands of paper documents… (GP7 Round 1)  * I flag it as ED that comes back to us, to say that an entry has been made by ED Westmead or Blacktown…like flagged in the patient’s notes here that the patient was in hospital on this date (PN3 Round 2)  * make sure if there is some kind of alert… so that everybody knows what's happening. (GP6 Round 2)  *Then we can evaluate the service in a very efficient manner. If everyone is on the same system and I think evaluating the system, you can crunch the numbers easily, compared to when everyone was using different databases and different sort of outcome (HS6 Round 2) |
| **Shared Patient Care Plans** | |
| **Implementation / delivery**   - *GPs usual care plan - different to proposed by WSICP* - *Nurses upload GP plan to Linked-EHR* - *HealthPathways as a means of providing GPs with the Shared Care plan protocol* - *Hospital staff cannot see Care Plans* - *Incorrect information in care plans* - *Care plan set up from first hospital visit* - *Delays in GP enrolment impact on value of care plans* - *Lack of GP understanding of functionality of Care Plan* - *GPs not alerted when changes made to plans* - *Some carers note their information is well connected and shared* - *Patient focused and directed* - *Importance of payment for development of the plan* | *the notion that we have one document that is a shared care plan between us and GPs is not – hasn’t reached the reality yet (HS8/MG8 Round 2)  *what my nurses are complaining, that we use the care plan per patient and Best Practice software that we use. And the integrated care program is using a different template so they are basically creating two different care plans (GP13 Round 2)  *Basically we just do our normal care plan for the patient that gets uploaded onto Linked-EHR and in our practice it’s going to be the nurse who is uploading that information to Linked-EHR. (GP5 Round 1)  *what I’ve also done in HealthPathways is I’ve organised this shared care plan protocol …So each time one of these integrated care patients comes in there’s a set thing, set protocol for how to. (GP5 Round 1)  *most of them are not even able to upload shared care plans because the system’s not even working yet. So the hospital can’t see it…All the care facilitators can see it, but the hospital specialist or hospital team can’t see it. (CF3 Round 1)  *We can’t access the shared care plans through – integrated care’s supposedly got – through the server we can actually link in with the EHR (HN4 Round 2)  *once the IT has finished, that they’re building, we’ll be able to, every time we see a patient, update the care plan and what my recommendations are and what my plan is, which I think will be a good thing that is promoting communication amongst the team(AH2 Round 1)  * when we see someone for the first time, we’re setting up a shared care plan and when we have case conferences, and then we feed that information back to all the relevant parties involved. (AH3 Round 1)  *I think we’re still struggling at the level of GPs understanding what a care plan is…what a care plan entails for ongoing management of these patients. .. that care plan through Linked-EHR can be shared with an allied health provider, a podiatrist or someone else but at the moment it’s not shared necessarily with the hospital clinician. (MG6 Round 1)  * it doesn't tell me who has added stuff to it, but I don't think anything has changed at this stage, but - actually maybe - yeah, I don't know if something has been changed or not (GP6 Round 2)  *One particular program that the GP put us onto, the fact that they managed to link my Mum’s entire medical history with both Blacktown and Westmead, through their GP (PC17 Round 2)  *all the focus on the patient, now he does like direct the care plan. (PC18 Round 2)  *often the WentWest chronic disease management nurse preparing the care plan…the GP is not even wanting to log on and connect to the EHR and see the care plan because they know someone’s done it and that they’ll get the money out of the care plan. (CF3 Round 2) |
| **Use**   - *Limited use by HS* - *GPs not aware* - *Good uptake* - *Not linking with hospitals* - *Not being updated with action plan information* - *Uncertain if the information is accessible or used* - *Enhances team care* - *Summarises current status* - *Not useful for referrals* | *so I’ve done care plans a lot with patients prior to this and I have seen what it looks like on the Linked-EHR so I know what it looks like, I haven’t actually done a care plan on Linked-EHR but I can understand what it is. (HS9 Round 1)  *Some people would argue in primary care there’s good shared care planning happening between primary care and allied health providers…I don’t believe it’s happening yet with the hospital facilities…they all should be part of the one team. (MG6 Round 1)  * The doctors here, if you said to them you can find so and so’s care plan, or whatever in Linked-EHR, the first thing they’ll say, “Is what’s Linked-EHR?” (PN2 Round2)  *It’s working a lot more efficiently with care plans. I think, 90% of my patients have a Linked-EHR care plan … (CF3 Round2)  *Care facilitators access yes. The hospital side, no, we might not be able to yet. (CF4 Round 2)  *We haven’t had any shared care plans that we’ve had to look at. (HN2 Round 2)  *I haven't seen much back or being added to the care plans as yet (GP6 Round2)  *I'm not entirely sure if they [specialists] are reading it or if they are making any comments because our nurse usually uploads the care plans once the patient is linked up with the Integrated Care Program, but I haven't seen any comments. (GP4 Round 2)  *the hospital clinicians still send an action plan to the GP but often GPs don’t really update the care plan with the action plan, because that’s how it’s meant to happen. (CF3 Round 2)  *our care facilitator, he’s excellent, he’s then documenting in the patient’s notes, he’ll ring me and tell me, “I’ve seen so and so,” and he writes in the notes, and that’s actually quite useful, but the doctors don’t actually look at them (PN2 Round 2)  *that was supposed to be one of the cornerstones of how we communicated amongst ourselves and with the GP, but the reason we’re not utilising it is because it actually doesn’t go anywhere and people don’t look at it and so we need to know if that’s up and running, so that we can start using it (HS3 Round 1)  *For us that’s only read only, so the GP is the one that alters that; we can see it, but currently not working in Cerner and can’t actually see the patients that are enrolled (HN4 Round 2)  *We haven’t had a lot, we’ve had a couple of patients that we’ve had multidisciplinary approaches with, with the endocrine, not so much with respiratory here. So we’ve had combined care plans for those patients, they seem to work okay. (HN2 Round 1)  *I did read the notes of the care facilitator on Power-Chart, which is helpful in terms of summarising their current care plan, and so I know where the patient is in terms of their care plan and where we can add to it (HS6 Round 1)  *rather than unloading to a shared care plan, I just send my care plan electronically with the paperwork through the podiatrist. He will electronically send me back a report … then I put that into my care plan for when do I do the next review… (GP7 Round 1) |
| **Experience (positive)**   - *PHN assists general practices with care plans* - *Sharing with allied health* | * WentWest helped us understand the care plans and how to review and upload and this has made a big difference to our practice’s efficiency and patient care (GP8 Round 2 )  * I do really like the ability to use the care plans or to share the care plans with allied health professionals.(GP2 Round 1) |
| **Experience (negative)**   - *Much time uploading* - *Practice staff not notified of care plan changes* - *GPs unfamiliar with Linked-EHR* | *our nurses have been uploading them onto Linked-EHR and it takes ages - quite a long time for that process (GP6 Round 2)  They [GP] say it’s all there on the system, the patient tells us their doctor has made a care plan and we can’t find it-makes you look a bit silly (HS4 Round 2)  * Resistant, I don’t know whether that’s probably the correct word to use. It’s probably more apprehensive about it. They [GPs] don’t like the change and new ways, especially using Linked-EHR. Linked-EHR is like a barrier to it, because it’s just time consuming, and the time that they don’t have (PN3 Round2) |
| **Satisfaction**   - *Ease of use* - *Effective communication of current status* | *Yeah, it’s easy to use as well. (PN1 Round 1)  *I think that was easy…is helpful so I know where the patient is in terms of their care plan and where we can add to it (HS6 Round 1) |
| **Perceived value**   - *Access to shared care plan central to WSICP effectiveness* - *GPs value as tool for team care* - *Concerns about volume and value of information* - *Sharing across hospital disciplines useful* - *Increased work may not be worth effort* - *Provides useful information for P/Cs* | *a linked in piece of the puzzle that I felt is probably the most critical bit for this program (MG3 Round 1)  *They’re going to be a really good resource for the GP, for the allied health, for the specialist, because it’s going to be up on Linked-EHR, everybody’s going to be able to access it (GP5 Round 1)  *I do really like the ability to use the care plans or to share the care plans with allied health professionals. I think having that is really useful.(GP2 Round 1)  * I worry that I’m going to be overwhelmed by the amount of stuff that we could access from general practice but I don’t want to access a pile of stuff, I just want the useful stuff (HS2/MG9 Round 1)  * if they’ve [patients] been seen in Cardiology Rapid Access or Diabetes Rapid Access, then I see that on the Shared Care Plan, and that’s useful. But in proportion for the amount of effort that has gone in for generating the Shared Care Plan to the usefulness, I think we could have spent our time doing other things. (HS2/MG9 Round 2)  * I think it’s a good idea. It’s - because they know what’s going on. (PC12 Round 1) |
| **Suggestions**   - *Use of shared care plan to facilitate communication* - *More use by hospital specialists* - *Greater patient centredness* - *Training and set up required* - *Streamline process of updating and uploading* - *Practice staff need to be notified of changes being made* | *… more communication going on through the shared care plan… referrals going straight through and everybody can work on the care plan together. (GP6 Round 1)  * I don't know how much involvement the specialist has in the Linked-EHR, but their ability to access the shared care plan would be helpful (PN4 Round 2)  * it should be more patient centred. That’s actually one of the things from the US, when you put in the goals for these patients – for these care plans you are generally putting in what the GP thinks the goals are but we really should be getting more what the patient’s goals are…GP5 Round 1)  *It’s just that it’s not an easy set up. It requires a physical engagement, enrolment, usually taking WentWest’s staff to do it. It requires some degree of training of the end user… if we had to tweak anything else in the end at the time that would be the goal, to make that actual shared care plan a much easier device to implement and to operate.(MG5 Round 1)  It is a very complicated way now, how the nurse and the care facilitator are doing that [updating and uploading], there’s some time, maybe half an hour, or something like that (GP11 Round 2)  * we uploaded them but I did it for the doctors [GPs] and they can view the results – the care plan online. They still need more education though, the doctors (PN6 Round 2)  * I'm not sure if there is an alert system or something like that so that everybody knows what's happening with changes (GP6 Round 2) |
| **Specialist Action Plans** | |
| **Implementation / delivery**   - *Plans more succinct* - *Limited implementation and isn’t sent electronically* - *GP unable to access Action Plan in power chart* - *Cannot access Action plan through Linked-EHR* - *Discharge summary contains recommended actions* - *Source of communication especially in rapidly changing clinical situation or complex cases* - *A source of patient information* - *Format not as well suited to some clinical situations where assessment findings need to be included* - *Labour intensive* | * They were getting letters from me before, now they're getting it in a more succinct way, and hopefully those that are already on board can look back and see the updated plans. (HN4 Round 1)  *I’m hearing that it’s not well implemented yet. it’s…. printed and given to the patient and emailed to the practice (MG5 Round 1)  *So, the issue with that at the moment, it still can’t be sent electronically. (MG6 Round 1)  * so the letter back to the doctors is still by paper mail…we can’t ensure that they’re on the common system. (HS1/MG7 Round 2)  * we would write an action plan, but that’s not functional at this stage. We write a letter and send that to the patient and their GPs. It’s mailed out to them, currently. That’s really the way that we communicate with patients and their GPs. (HS6 Round 2)  * what next will happen with that database, it will export the letter, but it also will automatically put a patient summary into the action plan on Cerner, and that’s what in the future the GP will be able to see straight away (HN2 Round 2)  * we can put it on to our power chart… So the GP does not have access to add to that or change that. So it’s fine for within the hospital but because the major person we’re communicating with is the GP, we haven’t been utilising it. … The main information is in the letter that you’re posting to the GP.(HS3 Round 1)  * we’re just getting it paper wise, they’re not coming through on the care plan, or the Linked-EHR, we have to constantly ring and ask them to fax it. (PN2 Round 2)  * Some of them, I do get electronic health patient summary …Sometimes I don't get anything. (GP13 Round 2)  * they send the discharge summary, always got follow up and, you know, see GP in two or three days, do this and then follow up with the specialist in six weeks (GP9 Round 2)  *Through the program we always write back a, management plan. So it’s not just about, how you’re going, and medication. I think, we haven’t actually been doing this very well at this stage but hopefully this will improve – to provide a management or an action plan so GPs now know how to tackle the more difficult, complex cases. (HS10 Round 1)  * there is a line of communication open there, but the main one really is the setting in respiratory and the steroid patients which swing up and down like a yo-yo, and we don’t know what they’re doing in terms of their full dosing and their potential outcomes so that has been a line of communication that’s been working quite effectively. (HS5 Round 1)  * I got a patient’s copy from the hospital with an explanation of what’s happened. (PC9 Round 2)  * every time I see a patient I have to do all of the assessment to then generate an action plan so, and then put that into a letter type format. So there is a lot of tweaking and a lot of, I think, extended writing in the cardiology format, when I see might four patients in a day, it can take me a day and a half to do the paperwork, yet, within integrated care all I’m meant to do is send out an action plan. And I don't think that I can honestly just send out things like, “Continue medications,” or “This is reduced,” without giving them the reasons why, giving them a set of obs. (HN5 Round 1) |
| **Use**   - *All hospital team provide information* - *Standard instructions that are reinforced with patient* - *Clinician and patient plans in patient focused language* - *Patient follows instructions but needs to see GP for prescriptions* - *HNs following up* - *GPs use to follow up* - *No feedback as not seeing patients* - *Offer selectively to patients who have good understanding* - *Unsure of clinician preference over discharge letter* - *GPs prefer summarised information* - *May not be appropriate or safe in complex patients* | * We all collaborate and put our few sentences in, and the specialist puts a letter together that goes back out to the GP, and out to the patient themselves (AH6 Round 2)  *ours is more general, like if this happens, seek medical help sort of stuff; it's a standard thing they get given anyway, so we just reinforce that when we meet them and say, “go and see your GP about this,” or whatever. (HN4 Round 1)  *so the patients that I have sent in they have come with a letter that has got a cardiology action plan, it's got like four steps in it, like that's good, and also things for the GP to do, and then they have got a patient plan… there were quite a few things on there that were sensible. (GP6 Round 1)  * Sometimes I’ve typed them out for the patient, or I’ve done all their medications out for them. Now, with the action plan, we can type it in and just print it off, and at least then they’ve got it for the GP as well. (HN2 Round 2)  * they give you an emergency plan. Like if I can't breathe, we'll start off on Prednisone and antibiotics and then you're supposed to go to the doctors. Well, I just take the Prednisone. I know myself if it’s going to be a hospital visit or a couple of days in bed (PC8 Round 2)  * I actually handwrite on a piece of a paper the instructions to the patient and give it to them, but whatever I handwrite and I write down the medical recommendations and the action plan so the GP and the cardiologist get both of them (HN5 Round 2)  * normally in the clinic, doctors print out the paper and bring it to the patients and for a GP too (AH1 Round 2)  * From the Breathlessness Clinic we do it a bit differently. We send a letter to the patient, addressed to the patient, in patient focused language. But that we send a cc to the GP and to the consultant. (HS2/MG9 Round 2)  * because the patient is the most important thing to us, we write the letter to the patient, and we put it in to layman’s terms, so the patient understands what’s happening (MG6 Round 2)  * And when you break down you’ve got to refer back to the action plan. They normally put in there two types of medicine, two types of antibiotics. If it doesn’t work, try this one. So the doctor also knows – actually she is the one that at the end of the day that’s got to give you the prescription for your medicine. (PC2 Round 1)  * I had a couple of patients who were profoundly constipated so referred them back to their GP and suggested giving them something like lactulose which they did and I then followed them up on the phone to see whether or not there’d been any effect from the treatment… (HN5 Round 1)  * that information flowing through to us when they've been admitted or discharged helped us get in contact with them and make sure that we follow up with them after that acute episode. (PN4 Round 2)  * We’re filling it in and we print it out, fax it off … but I don’t get any feedback about it. I think it’s a useful idea and I think it would be helpful for my GP colleagues but … I’m not getting any feedback about it. (HS2/MG9 Round 1)  *… you just have to make sure that you give it to the right patients who have a good understanding of their disease process and can identify when they’re becoming unwell. Because, you don’t want them to be abusing antibiotics and steroids. (HN3 Round 1)  * We should be testing well, did they [GP] actually look at it, was it useful, quality information and did it in any way inform practice, is it something they’d like to be receiving into the future and then there’s the thing around well, how is it different to a discharge letter. (MG6 Round 1)  * the letter is more comprehensive, so the action plan will only be a couple of lines. (HN2 Round 2)  * One guy [GP] goes, I chuck everything away except the first page because that’s where you’re putting all the information (HN5 Round 2)  * not everyone is safe to have a specialist - - - COPD action plan. For instance, if the patient has severe COPD, a lot of comorbidities, a lot of heart problems. If they become short of breath, it could be anything, including COPD. So it’s dangerous in that situation to give them an action plan. …So those kind of patients, I encourage them to present straight to the GP or hospital. Some patients who are, young and well educated, motivated, only one single disease or two other, you know, minor medical issues but the dominating issue is COPD and they’ve got family support, I’m happy to give them action plans. (HS7 Round 1) |
| **Experience (positive)**   - *Carer reassurance* | *I found the letter they gave us when I brought [husband] home reassured me in that I was already doing the right things and helped me so much in planning for other things that could help him (PC22 Round 2) |
| **Experience (negative)**   - *Accurate action plan takes time to write* | * it can take me a day and a half to do the paperwork, yet, within integrated care all I’m meant to do is send out an action plan. I don't think that I can honestly just send out things like, “Continue medications,” or “This is reduced,” without giving them the reasons why, giving a set of ob’s that they’re hypotensive. So the action plan embedded in the assessment does cause me grief (HN5 Round 1) |
| **Satisfaction**   - *CF helps implement* - *Improved patient care when patients part of the process* - *Summaries high quality and prompt* | * this is where the GPs are saying they love the process, because the care facilitator then is taking on, as we expected them to, some degree of responsibility for ensuring that the action plan has got the right and adequate lines and resources(MG5 Round 1)  * certainly improved their care and they’ve been very happy with the discussion had and time to understand it. (HS11 Round 1)  *We try to hand it out to the patient, taking it to the GP and they quite like that (HS5 Round 2)  *the discharge summaries have been coming out really promptly and really good summaries – good communication from the hospital about that (GP5 Round 2) |
| **Perceived value**   - *See what others are doing* - *Assists patient management and coordination* - *Assists patients if they act on recommendations* - *More effective than letters* - *Seeing evidence of use of the plans* | * At least we can see now a bit better what other people are doing, so I think that’s good. I think that’s a positive thing. (HS1/MG7 Round 1)  * It’s actually very thorough…it actually highlights them of some other stuff that wasn’t seen by the GPs, or the other specialist, and she actually gives a very simplified version of exercises that they need to do which actually helps patients in the long run. (HN6 Round 1)  *if patients are discharged from hospital we can read through it, work out if anything needs to be done before the patient comes in so that can save a little bit of time, and it helps with better coordination and management in terms of linking up with specialists as well. (GP4 Round 2)  * they sent a bunch of recommendations for us– I think they were wanting a dietician, wanting a physio, a whole bunch of things, which the patient agreed – said that he wanted, but we looked at his plan and we’ve done all and he never goes. (PN7 Round 2)  * I feel once it’s done it’ll be effective, because at the moment the doctors are just writing the standard letters.(AH2 Round 1)  *there’s a couple whom we’ve provided with, say, COPD action plans and they’ve sort of come back and they’ve used their action plan. We’ve got one in particular who’s really quite, you know, confident with his action plan and knows when to use it and knows when it’s not working (HS9 Round 1)  * like the Action Plan, it just gives you information – when you’re feeling good, what do you do when you’re not so good, and when you’re really bad – ring up the ambulance. (PC11 Round 1) |
| **Suggestions**   - *Clear instructions* - *Ensure shared with patients and with GPs* - *Electronic delivery* | *Make it as basic as possible. Saying, are you short of breath, take your Ventolin. If you start to cough up green stuff, take this and make sure that that plan is given to the patient, is given to the GP as well so everyone knows what the plan is.(GP1 Round 1)  *in the future, we are hoping that the action plan will actually go directly [electronically] to the GP, a consult, so they know what the plan is, from us. (HS6 Round 2) |

| **GP Support Line (hotline)** | |
| --- | --- |
| **Implementation / delivery**   - *Aspirational* - *Not well promoted* - *GPs not aware they could use for non-enrolled patients* - *Poor hospital staff orientation and awareness but some hospital staff are promoting* - *Not answered/ diverted to correct person* - *Work load for registrars* | *So the idea is that there’s hotline phone that the GP can ring and get some advice from me and then we get them into a Rapid Access Clinic so, you know, I talk to the GP. I say, “Okay, you’ve started them on their action plan. Great. I’ll see them in Rapid Access Clinic tomorrow.” And it’s another measure that’s meant to prevent admission (HS9 Round 1)  * It has been used on occasions by some GPs who stumble across it on health pathway - on the WentWest website – other than that it’s not been openly advertised to GPs (HS5 Round 1)  *we hadn’t realised initially that we could ring up - patients who were not involved in integrated care, we could still ring the GP support line. (GP5 Round 2)  *… lack of awareness that they’ve [GPs] got that service that they actually can use. Not to say that we haven’t had GPs ring up…but it’s few and far between (HN2 Round 2)  *Now we have a GP support line and the numbers are all on the HealthPathways website (HS6 Round 2)  *The problems that I've had is the registrars that don’t know what's going on. I've had to make three or four phone calls …different registrars – they don’t know what's going on. (GP1 Round 1)  * respiratory CNC and myself did a talk to community nurses, so when they feel that there's someone that’s sick enough out there they are also calling us and I’m automatically telling them they need to get onto the GP to call the GP support line. (HN5 Round 1)  *two GPs in particular…they're either being fobbed or no one is available or they’re answered or the person who's answering doesn't really know what it's all about. So at this stage we've got a lot of glitches (MG3 Round 1))  * That has changed. The registrar’s now when I ring up, they totally know what is going on and it’s a bit more streamlined. Yep, it’s a better process. (GP5 Round 2)  *it’s meant to work to 7 pm at night, the GP Hotline, so if it’s not answered after 4 o’clock the phone’s not diverted anywhere so they were going to fix that. (CF3 Round 1)  * … last month where a GP had rung at 5.30 or 6 o’clock and that’s when ED rings the advanced trainee to offload the ED patients. So he was on the phone sorting out five or six consults, could hear the messages coming through. As soon as he got off the phone …he rang the GP back immediately (HN2 Round 1) |
| **Use**   - *Underutilised but some GP registrars are using more often* - *Use improving and CFs utilising* - *Fast admission to RASS* - *Some GPs time poor or reluctant to collaborate* - *ED using more than GP* - *Inappropriate use – not for designated illness* - *Value in empowering GPs* - *A good source of advice* - *direct communication with specialist/ registrar is sometimes used instead* | *we've had very little uptake from GPs at this point.(HN4 Round 1)  *It’s hopeless. I think that the GP Hotline is not used because the patients that just don’t go to their GP when they get sick. They come straight to the hospital. So the GP doesn’t have an opportunity to intervene. (HS2/MG9 Round 2)  *the trainees who take the call, they’ve been seeing very few heart failure calls. (HN4 Round 2)  *we’ve been a bit disappointed by the hotline. We’d like to be able to have that utilised but don’t feel that it’s being utilised as much. (HS1/MG7 Round 2)  *we've had good feedback, especially with the amount of patients that I book with regards to the referral and coming from the GP… it's actually picked up (HN6 Round 2)  *I haven't - not this year - actually my registrars, they use that a lot. And they find it really useful. (GP9 Round 2)  *GP support line calls are improving. Not a huge amount, but definitely more than before. I'm getting calls from GPs…The community nurses [CFs] are calling me quite often as well… (HS7 Round 2)  *the only time I ever used it was like, I just rang up and asked to get someone in and that happened, but haven’t had to actually to go through anyone - discuss the patient with anyone (GP14 Round 2)  I think just they’re very busy, they’re not familiar with it, until they’ve tried it they don’t realise how helpful it is, and sometimes they don’t want to let go. The GPs can be very precious with their patients sometimes as well (HN4 Round 2)  *they have been utilised by ED ringing up, but that’s – it was meant to be a GP hotline (HS1/MG7 Round 1)  * it takes time for a GP to understand how to use it, because some of the calls have not been appropriate. (MG1 Round 1)  *they do get calls about other cardiac problems, which of course they deal with, but they don’t meet integrated care criteria. So they don’t actually come through the RASS clinic. It has to be chronic illness heart failure. (HN4 Round 2)  *I get an average of maybe one call a week, maybe two…unfortunately those calls are more for general advice about things to help run their day-to-day sort of patients, for example questions about the medications… not related to what this integrated person is about (HS10 Round 2)  *…a GP just found the number and had a question about someone with lack of thyroid. And I can see how it’s a really, really good idea, again, empowering the GP…building their skills and again giving access to endocrinologist. (AH2 Round 1)  *...I found it quite useful, getting some advice. And sometimes we don't even need to send to the hospital, just talk to the specialist, and get some advice and then if they do need to go in there - they might just see the doctor at the clinic and then come back the next day for review (GP12 Round 2)  *… because of the work we've been doing with case conferencing I get half a dozen calls a week from GPs and so does my registrar. We're trying to divert that to the hotline but it's hard to change (HS8/MG8 Round 1) |
| **Experience (positive)**   - *Limited experience of working well* | *from what I’ve seen, it works quite well from the experiences that I’ve heard. (HS11 Round 1)  *I think GP hotlines are working well… The GPs that have called in have gotten good responses. (CF2 Round 1). |
| **Experience (negative)**   - *GP support line not answered/ operational* - *Hospital staff not orientated* - *GPs perceive differences in activities between hospitals* | *feedback has been the frustration with the times when the GP support line hasn’t worked properly. It’s not been answered or it’s been answered by someone who doesn’t understand the program(MG5 Round 1)  *I had a GP who I encouraged to use the hotline when their patient was unwell and when they called the hotline it basically said this number doesn’t exist [laughs]. They had to choose another option, like we called again so finally we were able to be put through to a department and then they said, “You’ve rung the wrong department. This is not cardiology,” and they gave a mobile number to the GP to ring the registrar that was working for that day. When the registrar answered the phone, the registrar said the Integrated Care Program is not live yet.(CF3 Round 1)  *I was ringing up about an ischemic heart disease patient and because I rang to Blacktown they weren't set up to manage that there, they said Westmead's team is but Blacktown's wasn't, so they just advised to send the patient to the ED rather than use Rapid access which is fair enough. But it seems like there was still some differences between what the two hospitals were doing (GP6 Round 2) |
| **Satisfaction**   - *helpful responses* - *Good advice* - *Also unsatisfactory responses* | *I've rung it a few times…everybody that I have spoken to so far have all been cardiology and one was really good. … they have all been quite helpful and good to speak to (GP6 Round 1)  * The GP support line. I haven't used it more than once but it was good (GP13 Round 2)  * So I’ve used that GP support line. I’ve found that quite helpful … (GP5 Round 2)  *That's been great, so I've used the cardiology and the respiratory and the endocrinology team for advice and that’s been good. (GP2 Round 2)  *…the person who had the original pager had given it to someone else at the hospital, the doctor wasn’t very impressed with that, because, that was the first time she used it…it’s a lot of running around and chasing up and she couldn’t get hold of anyone (PN2 Round 2) |
| **Perceived value**   - *Helpful in avoiding hospital admission* - *Source of information – consultation especially for new GP registrars* - *Can provide pathway into hospital services e.g. RASS* - *Direct access an advantage* - *Link to Rapid Access* | *if there is any change of the patient you don’t have to send them into the emergency, you can call the hotline, talk to the team, they can see the patient on the same day or the second day. (GP3 Round 1)  *the link it provides to GPs, the GP support line, is another way of preventing admissions. Historically, if GPs were unsure of these patients, they would just say, “Go to emergency,” but if they have something to fall back on, again, that’s helping out the community in terms of building a bridge between primary care and the specialists. (AH5 Round 1)  * Anything that bypasses emergency and is subacute that can be treated with access to the hospital facilities without going through emergency, is going to benefit everyone. Yeah, so I found it quite useful, getting some advice. And sometimes we don't even need to send to the hospital, just talk to the specialist (GP12 Round 2)  *I’ve seen so many new registrars being trained for GP practice and they don’t know how to do things right – and they’re not all that sure about how to manage the patient, so these GP support lines have been very valuable support for them with those kind of patients. (CF4 Round 2)  *… in the past the only access us GPs had to the specialist, if the person didn’t have a private specialist, was to ring the registrar on call and they always didn’t have time to have a bit of a chat with you about the slightly more complex management of diabetes through medication… whereas now that GP support line is a really good access point for the GPs to get some information… without having to send the patient to …hospital (GP5 Round 1  *It’s actually great because, yeah, they have, like, Rapid Access and the doctor doesn’t need to, you know, look for a directory just to have an access in the hospital. (PN1 Round 1) |
| **Suggestions**   - *Poorly promoted* - *Promote widely to GPs early on* - *Use patients to promote to GPs* - *Extension to other disease areas* - *GPs need to be aware of the types of services that can be accessed* - *Hotline access contingent upon relationships* | * I think there could have been a little bit more advertising and communication of who are the people at the end of the line. (CF1 Round 1)  *the first thing they should have done was try and get as many GPs as they could on board. (HN3 Round 1)  *We had one incident where it wasn't manned and somebody at Westmead actually said the lines not operational. So I think there needs to be awareness that the program is up and running (CF2 Round 1)  * our patients that we have enrolled in Integrated Care, we give them the information about the GP hotline, so when they’re going to the GP and their GP is struggling or not sure of what to do, they say, “Don’t forget we’ve got this hotline.” (AH3 Round 1)  * So with this rapid access line, because my referral was not for ENT or orthopaedic, it was for diabetes. So it worked, but if it was for ENT, eye or orthopaedic, will it work, that's my question to you? (GP13 Round 2)  *Maybe a bit more aware that the GP, so they know who to call for what kind of thing (GP6 Round 2)  *I get half a dozen calls a week from GPs and so does my registrar. We're trying to divert that to the hotline but it's hard to change, and it's because of the relationship. So the hotline would get hot if the relationship was built (HS8/MG8 Round 2)  * if the patients are being risk stratified within 48 hours, I think that’s a very good thing, and it’s really a safer approach in terms of managing ischemic heart disease. So I guess that’s the relationship we have with emergency. I would like to see in future a similar relationship built with the general practices. I mean, we only get maybe one or two calls a week from the GPs around this area, and I guess that’s something to improve on. (AH5 Round 1) |

| **Rapid Access and Stabilisation Service (RASS) Hospital Clinics** | |
| --- | --- |
| **Implementation / delivery**   - *Communication challenges make KPIs difficult to achieve* - *Utilised mostly by ED in some specialties* - *Poor GP engagement* - *Fast track patients* - *Multidisciplinary clinic* - *Thorough* - *RASS is integrated with existing heart failure services* - *Provides outreach* | * we don’t have e-referrals, so you get a fax that turns up on a fax machine the day after the patient’s visit, and then you try to ring the patient and they’re not there, and you ring the patient again and they’re not there, your KPI of two days is gone. (HS1/ MG7 Round 1)  * when the patients go and see the GPs, the GPs would ask, okay, if they haven’t got a letter back from RASS clinic, the GPs will ask the patients and sometimes the patients cannot remember (CF3 Round 1)  * Most of our patients are from ED, and so I guess it helps out emergency in that all these patients … would otherwise be admitted or be in short stay, or just have a prolonged stay in emergency before they have a discharge destination, or a plan, but the rapid access clinic provides them an option for early discharge (HS6 Round 1)  * I haven’t accepted a patient or have been referred a patient to the clinic from a GP as of yet. (HS10 Round 1)  * So they fast-tracked me and they took me straight up to the ward. I didn’t have to go through the emergency care, wait there for about four or five hours, none of that. So they go, that’s what happens with Rapid Access. (PC2 Round 1)  * When I go there's pretty much– as far as the heart clinic goes, I'm pretty much straight in. Not sort of sitting there or waiting around for hours and hours (PC4 Round 2)  *there’s still room to improve but it’s become much more a rapid access service. So, either from the GP or from emergency – I think that’s running a lot better, and the processes of getting them in are running a lot better (HS3 Round 2)  *I think it was good. The patient did get an appointment in a clinic within two weeks. (GP13 Round 2) *having a nice multi-disciplinary clinic has been really good, because one of the challenges with diabetes is that it’s not just the doctor bit, you have to have the diet, you have to have the education. .. So we have a conversation together, pick up different aspects of the review. (HS3 Round 1)  *she’s [HN] very thorough actually. She didn’t want me to go until I’d done everything, she was – they looked after me well, to tell you the truth. (PC11 Round 1)  * it is fully integrated into a heart failure service –it’s one heart failure service that has the existing heart failure services for in-patients, stabilised clinic patients, home visits, phone follow-up, and people from integrated care come into the rapid access service, for stabilisation and when they’re finished there they’re referred to the heart failure service for ongoing follow-up (HN4 Round 2)  *part of our clinic is a breathlessness clinic where we do a lot of outreach visits to patients. The aim of that clinic is to provide patient care at their home, because these patients are just too breathless to worry about coming in to a hospital (HN3 Round 2) |
| **Use**   - *Lack of GP referrals to some RASS clinics* - *RASS as a source of information and individualised care* - *Use by ED* - *Some GPs use specialists* - *GP use depends on their clinical capacity* - *Fast track admission from RASS* - *RASS used not only for enrolled GP patients* - *One stop shop* - *Inappropriate referrals from ED without medical check back* - *RASS does not exclude patients* - *Internal hospital use of RASS* - *Transition from Rapid access to stabilisation* - *Locating RASS at relevant clinical departments* - *Patients able to access as required* | * we're not getting a lot of referrals from GPs at this point (HN4 Round 1)  *GP referrals are steady but nowhere near as much as we get from emergency departments or any other service of the hospital.(HS6 Round 2)  *I don’t see a lot of GPs actively ringing the RASS service despite our promotion but, I think, sometimes, unfortunately on the negative side, sometimes we’ve had a few bad experiences as well in ringing RASS (CF3 Round 2)  *I think the service is being utilised more and more. But I'd say maybe 50/50 in terms of hospital referrals and GP (HS4 Round 2)  * they said “here’s all these programs and numbers, and brochures, and things, and you can do them if you want”, but I wasn’t really interested because I’m busy anyway (PC3 Round 1)  *we had a nurse, and a doctor, to do with the pacemaker and, not that they tell you much, they talk – between themselves, but I kind of gathered everything was all right, and they improved the medication, but, they seem to let you know what’s going on (PC19 Round 2)  *they're organising something for me to do some like exercise up there in their facility under supervision and stuff. That was a bit of a suggestion because I'm just a bit concerned if I try to do something at home and I have an incident that I'm stuffed (PC4 Round 2)  * I think, at this stage the main thing it is being used for is if someone comes straight through to ED and has a problem with diabetes, and that essentially is the main reason why they’re actually here then to do something to temporise the situation, and then to send them to be seen in the next clinic (HS5 Round 1)  * I’m getting referrals from ED, unfortunately they’ve all been inappropriate so it's been initiated by the nurse in ED who is not discussing it with the medical staff because all of them have been admitted. (HN5 Round 1)  * ED is getting good and quick and say, well, actually you don’t need to come to ED, just go to RASS. So, while we’re not actually completely bypassing ED certainly making the route through easy and much faster (HS1/MG7 Round 2)  * There's only one patient I can think of that I – try to avoid hospitalisation and referred to the cardiology … that worked out really well for them, but other times we tend to use specialists. (GP1 Round 1)  *I still think a lot of community heart failure patients have their own cardiologist they [GPs] prefer to send them to the cardiologists rather than send them to us (HS6 Round 2)  * respiratory is a bit of a waste of time, ‘cause I can manage a lot of the respiratory patients. One patient would have probably needed some home oxygen and the specialist organised that. I've got one of my patients that was an acute heart failure patient that I got someone to see her quickly and we managed to treat her that way. (GP1 Round 1)  * I’ve had the one specific example of a patient who was unwell of my own who I sent down and they were assessed in the rapid access clinic and in fact they were deteriorating so they had a facilitated admission which was very effective for that patient’s healthcare at the time. (MG5 Round 1)  * I was willy-nilly at first, because that’s the way I was before, through the revolving door - it’s a one stop shop. (PC2 Round 1)  *I think the large majority are appropriate, but there are some patients probably aren’t completely appropriate, but we see them anyway (HS1/MG7 Round 2)  *I also find the people that we don’t necessarily have enrolled in Integrated Care … we have them in our pulmonary rehab service and they get sick, we’re then able to refer them to the rapid access clinic, and get them seen that way and that’s been really efficient. (AH3 Round 1)  *some of them come back, because we go from rapid access to stabilization. So if you’ve come direct and you need a bit more ongoing care, then you move to stabilization. There may be a couple of visits, then you’re back to GP. (MG1 Round 1)  * we’ve elected to have integrated clinics on level seven in the respiratory area… so we have all the resources, and everything here (HS11 Round 1)  *I think that is the best thing because I retain CO2 and sometimes I’d sit there and I’d think, if I could just get my blood gases checked and if they’re low, terrific, if they’re high, I can do something about it before it gets worse and I feel like this clinic thing is really good for me. (PC8 Round 1)  * I just have to ring up, if he’s having problems I just take him up to the heart failure clinic and that’s it. Or ring up the cardiologist, and they just tell me to bring him in, or just take him to the hospital. (PC16 Round 2) |
| **Experience (positive)**   - *Comprehensive care* - *Friendly and supportive* - *Assist in self-management* - *Time efficient* - *Special (individualised) treatment* - *Good communication and support* | * Wonderful, they have been really wonderful. When I do go into the clinics for an appointment it's a friendly atmosphere, they do all they have to do to check everything with me and then they explain all the things that I either should be doing or could be doing (PC7 Round 1)  *there was a particularly little Welsh nurse, and she was fabulous. I felt as if I was getting support. (PC22 Round 2)  *he talks very highly of the program … now after having COPD for a very long time and panic attacks for a very long time, he was referred to that program and he has been helped by that (AH5 Round 1)  * it’s been amazing; she’s never had to wait and I was really concerned about having to wait that particular time in the waiting room setting – I’d come straight from school that day, so yes, I always thought that hospital was just fantastic. (PC1 Round 1)  *A comment from the one patient that I sent down to the rapid access was it was magic, it was gold, treated like special rather than waiting in emergency for hours and hours feeling unwell, here I was being seen by people who met me at the door with a wheelchair and took me places to assess me and then decided I was unwell and then admitted me directly onto the ward. So they felt like they were royalty as far as the process was concerned (MG5 Round 1)  * you’ve got to put the big tick for Rapid Access. The doctor, [CF], the whole three of them, really, they are all linked together. So without one of them not being there I dare say it could be very, very dark. (PC2 Round 1)  * He has had a very good experience dealing with the heart failure clinic, like the rapid access stabilisation clinic, so having that good communication and extra - the patient felt much more supported (GP6 Round 1) |
| **Experience (negative)**   - *Inconsistent information to GP* - *Risk that patients bypass GP* | *it’s different between different hospitals and different clinics and again the information that goes back to the GP is not very consistent amongst the clinics, which is a bit of a barrier (CF3 Round 1)  * I’ve had a patient call me and she’s a joint respiratory cardiology and she goes, “Oh, I knew that if I called one but it didn’t matter which one I called because I knew you'd make an appointment and arrange to see me.” And I’ve gone, “Well, did you see your GP?” “No, there's no point” (HN5 Round 2)  *our patients as soon as they get breathless, or they are sick for a couple of days – they don’t go to their GP, because …my GP is too busy, or they are just too breathless to make the trip…the easiest thing for them to do is call the ambulance. (HN3 Round 2) |
| **Satisfaction**   - *Life changing* - *Avoid admission* - *Approachable and helpful* - *Continuity with and familiarity with a team and with GP* - *Good patient communication and support* - *Rapid access to specialist services can improve clinical outcomes* - *Patients seeing benefit including with home follow up* - *Multidisciplinary management* - *Shared interdisciplinary perspectives* - *Patients appreciate RASS care* | *Well, it’s changed my life. Because before I wouldn’t go – I wouldn’t leave the house. Now I can. I can go here, I can go there. Yeah. Big change. (PC2 Round 1)  * there have been some that we've able to send home and just follow-up in a week's time, so I think they're working well. (HN4 Round 1)  * clinic staff were fantastic. Always approachable, always gave patients an appointment, followed up really well, liaised with me really well to take back to the GP. (CF1 Round 1)  * And you can ask them anything and they will answer you with a straight answer. I don’t know, they just help you a lot. (PC12 Round 1)  *They’re brilliant, I mean, they explained what will happen, and how to deal with it (PC14 Round 2)  * They’re good, because I know the people that work there. They’re very, very friendly. They make you feel, well I know I shouldn’t say this, but they make you feel welcome as if they really want to help you. (PC12 Round 1)  * I think from most of them we’ve had quite positive feedback in that we end up being a group of people that they see over and over again. So it’s the same people, yeah. And, you know, again because these are the sort of patients who often are in hospital a lot, we end up being the continuity. (HS9 Round 1)  * she [HN] understands me very well. I can tell her anything. She understands. (PC12 Round 1)  * He has had a very good experience dealing with …the rapid access stabilisation clinic, so having that good communication and extra - the patient felt much more supported, so that's been really good… not just out there sitting at home getting sick by themselves, that there are people who are actually going to be able to help them. (GP6 Round 1)  * they’re continually ringing to see how I am. No. I don’t think I can do much better. (PC14 Round 2)  * I’ve only been twice [to RASS] and each time – and I’ve had three visits from the nurses at home and I could not fault one thing. I feel very happy about it. (PC8 Round 1)  *They’ve got the community nurses that come around and check me out once a month. They're very good. They just reassure me that I'm doing the right thing. (PC2 Round 2)  * it’s helped me a lot in terms of getting them access aside from a hospital admission, to urgent care and, especially with diabetes, that’s all they need sometimes and then they can really turn around quite quickly. (HS3 Round 1)  * they’ve been able to see everyone else by coming to the same place and not then having to next week come again to the hospital, to see the dietician and go somewhere else to see the educator (HS3 Round 1)  * the multidisciplinary team that we’ve created for the breathlessness clinic has been really useful and it’s really interesting because we’ve got OTs, the dietician, a physiotherapist, a nurse and a psychologist on that team….having the opportunity to talk to other clinicians that are not doctors about COPD and get their perspective about how we might better manage these patients has been just gold. (HS2/MG9 Round 1)  *I’ve had a couple of emails [from patients] that have been very positive; thank you emails afterwards. That’s been really good (HN2 Round 2)  *they all love our clinic and they don’t want to leave at the end because they get very intense care (HS3 Round 2) |
| **Perceived value**   - *Prevent admissions* - *Quick access to specialist care* - *Early intervention* - *Good liaison with GPs* - *Multidisciplinary care* - *Cross disciplinary care* - *Good care for complex patients* - *Access to specialists in public system for those without private (e.g. low SES)* - *Provision of patient education* - *Patients see benefits of improved health* - *Encourages self- management* - *Improved post admission care* - *Ability to see outreach patients* - *Time efficient to see multiple providers in one visit* - *Faster hospital discharge* - *Benefits of time* - *Having time to spend with patients-time to work better* - *Time to educate patients* | * is of value because it has prevented some admissions to hospital. (HS4 Round 1)  *Before [HN] came along, I’d been into hospital two times in six weeks. (PC11 Round 1)  * but those three patients that I’ve had a walk in visit with would have probably turned up to ED otherwise, and we’ve been able to – between the GP and follow up with GP and then coming back to see us very quickly, probably have prevented their admission. (HN2 Round 1)  *what the patients really appreciate is that there is that step before having to be admitted to hospital, so if they feel that they’re getting unwell; one, they’re a priority patient in the practice so they know that they can ring up and we’ll see them that day; two, that they do have access to a specialist reasonably quickly (PN3 Round 1)  * there’s no such thing as waiting time with them. They are all – you go to your doctor, or I get onto a hotline to them, so they want you in there ASAP because they don’t want you to go to hospital. (PC2 Round 1)  * to have a rapid access drop-in service has been of most value. Just to have an ability to be able to give a patient something right away, or to do it tomorrow, and not have to worry about appointments and things (HS1/MG7 Round 2)  *I’ve had experience of hours in the waiting room like everyone else, but I think your system bypassed that and I am checked over in no time at all in the hospital bed (PC9 Round 2)  * Where can you go with 48 hours and see a consultant for free? I was just saying earlier, there’s nowhere. (HN2 Round 2)  * we’re seeing a fall in our unnecessary admissions and …we’ve been able to sort of pick them up before they’ve come into hospital in extreme need. (HS1/MG7 Round 1)  * the Rapid Access Clinics, have been able to get people through the door quickly and facilitate that approach and recommendations are made by the clinic conveyed to me by letter (GP1 Round 1)  * very good at getting communication flowing, she’s [PN] getting all the reports and feedback quite quickly and that's been really valuable so you know what's happening with the patients at all times (GP6 Round 2)  *they would see the diet educators and doctors all at once – so you can package the service into a one hour, two hour period, rather than, say, an admission or have a patient come back three times to see different parties (HS4 Round 1).  * see them with the cardiovascular team and the foot team down there, because they think that the diabetes may in some way be attributing to their foot ulcer and their foot wounds and that there could be an improved outcome if we improve their blood sugar levels …that’s one of the examples of rapid access which can actually be quite transformative. (HS5 Round 1)  * It’s very good for the complicated patients. (GP3 Round 1)  * if you're under the care of a specialist, you tend to refer them to – or you're going to call a specialist rather than call the RASS Clinic to look at them. For those that haven’t got a specialist, it’s an advantage. (GP1 Round 1)  * the clinic has been good, especially for patients who are not financially well off (GP4 Round 2)  * Like people have been connected to the rapid access clinic who have never seen a respiratory specialist for years because of a lower socioeconomic, now they’ve had a chance to see a specialist…Medications have been changed. Their lifestyle has improved. (CF1 Round 1)  * she’s become more amenable to psychological intervention after figuring out that it was more about breathlessness related anxiety intervention rather than anything else (AH5 Round 1)  * I’ve learnt how to control – if I’m having a panic attack. If I’m having a anxiety attack, which has been – they’ve been so helpful up there. And, just the general breathing part of it. (PC11 Round 1)  *They show me the exact things I should be doing. Like, I didn’t exercise properly. They’ve showed me how to exercise properly. They talked me into going into the rehab for my lungs…They’re very good. (PC12 Round 1)  * it has been really useful to encourage self-management amongst patients (CF3 Round 1)  * I think that more intense connection initially, will lead to regular service and help them self-manage a bit better. (HN4 Round 2)  * We find out that people don’t have their follow up appointments made, or they didn’t understand what was happening in hospital. We check their inhaler technique. I think that does a powerful amount of good. (HS2/MG9 Round 2)  * A different way of looking at heart failure. I think we cover everything as far as education goes, as far as follow-up goes, as far as support goes (HN2 Round 2)  *What do we call it – I’m trying to think off the top of my head – it’s been a long day, but Outreach, that’s what it is, so Integrated Care Outreach Program which I think is good because we get to see patients and we wouldn’t normally see around that area. (AH2 Round 1)  *The clinics have been useful, just having that quick access for the patients to be seen is really important, because the general practice setting and not have to just go to ED, I think actually there is a step in between now and that's really important (GP6 Round 1)  *I really believe in the rapid access and stabilisation clinic you really see the value of patients coming in, getting reviewed by the specialist care treatment team and then they go back out in the community.(CF2 Round 1)  * the heart failure service is quite valuable …because they can get follow-up very quickly once they discharge these patients from hospital and prevent them from coming back and being readmitted.(HS6 Round 2)  * you need a long time not just to cover their COPD but the osteoporosis and heart failure and reflux, and other things that could be heading towards it and do questionnaires and link them into rehab exercise programs. So I think they have been able to dedicate the time that these chronic, complex patients need which probably cannot possibly be met in primary care or even in specialist care because of the time involved and the general frailty and complex nature of the patient (HS11 Round 1)  *Because they spend an hour with us. Spend the time to explain everything and we understand so much better about what it was and action plan and how to prevent getting sicker and prevent coming back into hospital. (HS11 Round 1)  * we’re trying to spend a lot of time with them to try and increase their understanding and really make them understand the importance of self-management and trying to, keep in touch with us in the initial period after hospitalisation to try to prevent them from coming into emergency. (HS6 Round 1) |
| **Suggestions**   - *Referrals without GP approval* - *Focus on prevention* - *Collect long term patient data* - *Expand clinical criteria* | * it's set up so that the GP makes the referral, so if a nursing staff member from the community or the care facilitator sometimes rings us… then we have to chase to get a medical referral, so that's an issue... (HN4 Round 1)  *management is really more a long-term thing and we may not see the results of this until maybe five years or something… So it would be very interesting to keep a track of these patients to look at long-term. (HS6 Round 1)  *I think it would be good to expand it a bit, it would be probably good to look – well, we’re doing heart failure but to link in a hypertension clinic. (HN2 Round 1)  * if we could function as a mini clinic that is able to exclude acute coronary syndrome, then I think GPs would feel more comfortable sending the patients our way (HS6 Round 2)  * they’re looking at an arrhythmia part to the clinic as well which may possibly be one day a week, and that will be good as well (HN2 Round 2) |
| **Patient Hotline** | |
| **Implementation / delivery**   - *A means for direct patient contact reassurance and autonomy* - *A central clinic number* - *May provide a bypass for CFs* - *Phone always answered* | * the patient hotline at the moment has enabled them to contact us directly, so maybe there’s no need for a care facilitator (HS1/MG7 Round 1)  * we’ll give them the phone number of an educator, we say, please call us if you have any concerns, call us anyway in a week’s time so we can help with insulin doses or with what you’re doing (HS5 Round 2)  *She always said we could ring them up at any time. They made sure of that. (PC22 Round 2)  * we are now actually planning this year to start up a hotline where we provide probably a 12-hour service to our patients, so that when they are breathless, they don’t know what to do, they have someone that they can call (HN3 Round 2)  * we have one number that they call when they opt to – whether it be OT, physio, et cetera, and that works – so you don’t have to worry about what number to ring because seriously it’s hard enough knowing who to ring from my point of view, let alone their point of view (AH4 Round 2)  *But their number one rule is that if anything happens to you, no matter what time of the day it is, give them a phone call. They pride themselves on a 100% pickup. (PC2 Round 1) |
| **Use**   - *Patients call as early intervention* - *Ring clinic to avoid clinic attendance and hospital admission* - *Patients call or drop in when serious concern* | *And if they do notice that their symptoms deteriorate, they often have the number for the CNCs at the RASS clinics, so they will call the nurse practitioners or the CNCs to say this is what’s been happening. I can see my weight has gone up by this, so they’re taking a bit of early intervention rather than sitting at home and waiting for symptoms to get worse and come into hospital. (CF3 Round 1)  * I’ve got a direct line straight to Rapid Access. There was a couple of times I’ve been up there …if it’s serious and I feel that the medicine is not working.(PC2 Round 1)  * he used to come into… rapid access once a week or once every fortnight…I thought that he used integrated care well, because whether he felt unwell, he would actually call (HN3 Round 1)  * Like, I can ring up whenever I want, the thing is if I need anything – which I don’t go and ring up any old time, it’s only when there’s something really wrong with me, that I give her a ring. (PC11 Round 1)  * So we give them their numbers, we give them two phone numbers, mobile numbers of the educators who are here five days a week and they then can actually call me to inform me or send me an email about themselves and I get back to them in real time (HS5 Round 1)  * it’s more an interaction with our nurse specialist… basically it’s a hotline for them to call the nurses if they experience any problems, and to get input from them. (HN6 Round 1)  *I’ve had patients that I see in my normal clinic that have also been coming in to - been using the integrated care services, and they like having that in between. They feel like it’s a level of safety in that they can come and talk to as well. (HS1/MG7 Round 2)  *they carry a mobile between them and I just ring them anytime I need them (PC4 Round 2)  *they use it. And they appreciate that help is available. It gives them a bit more confidence when we're making big changes to their treatment (HS4 Round 2)  *I'd rather be at home than in hospital and if I have any problems I can ring my doctor, my GP, and I can also ring the clinics at the hospital, I’ve got names and phone numbers that I can contact if I have any problems. (PC7 Round 1) |
| **Experience (positive**)   - *Improve patient understanding of their condition and who to contact* | * I certainly see patients ring up on the hotline…our feedback from them has been that they are happier with their understanding of their condition and who they need to talk to. (HS1/MG7 Round 1) |
| **Experience (negative)** | * |
| **Satisfaction**   - *Patients understand their condition and know who to contact* - *Enables 24/7 review* | *… patients ring up on the hotline, so I think they feel that that’s better, and our sort of bits and pieces feedback from them has been that they are happier with their understanding of their condition and who they need to talk to. (HS1/MG7 Round 1)  * they have been positive about that, to have a name and a face to call back on, so to speak. (HS6 Round 2)  *the patient is really happy because he has the phone number of the rapid clinic – access clinic and he, over the weekend, we are not working and sometimes he just called the clinic and then straight away went there. Patient is really happy. (GP3 Round 1)  *we get phone calls from people that we have discharged you know for a long time because they’re worried about their conditions. I think especially the carers are happy. Because they don’t have to worry about going to GP (AH1 Round 2) |
| **Perceived value**   - *Ease of contact* - *Patients feel secure knowing they have contact numbers* - *Patient autonomy supported* - *Care in the community supported- ED presentation avoided* - *Patient education* | * be a designated person, point of contact. So, at the moment, if I go and see them, and the clinic psych goes to see them, the dietician sees them, there’s three people, with three contact numbers …there is one person that they can call to ask questions of – just general questions, instead of going, “Oh, who do I call?” (AH4 Round 1)  * a patient we saw a few weeks ago, is not getting better and the GP is not happy and so the patient rung and we said, come in right now. So, we were able to attend that today, and that patient had a core line which was our integrated care CNC and she was able to come in, and prior to integrated care, we wouldn’t have had that sort of connection (HS1/MG7 Round 1)  * giving that patient the ability to have contact people in the program… that gives that patient extra… reassurance, and autonomy as well, they know where they can go if something is wrong, not just here to the GP, but they also get that extra knowledge that there is a heart failure clinic … somebody will see me; (GP6 Round 1)  *it’s like a safety net…if anything were to go wrong … they may present to hospital for something minor that could have been sorted out as an outpatient (HS6 Round 2)  *if anything untoward happens, I know that I can get a hold of her straight away, and she can then say, “Get yourself up here,” and I just put him in the car straight up to the heart failure clinic. (PC16 Round 2)  *Well, now I can just ring up and say I need to come in. I come in and I take my blood gas and see what my oxygen levels are …Once they wouldn’t let me go home because my oxygen was so low and the next time I was terrific. (PC8 Round 1)  *I feel not so scared or not so – I have to wait and think will I go to hospital, won’t I, will I, do I ring the ambulance, what do I do. I can just ring the girls up and say I don’t feel very well and they’ll say come in and I can see them without hospital. (PC8 Round 1)  *So it seems to have changed because she got access to everything. And then if she is not too happy, she just phones Rapid Access and tells them that I’m on my way up. But I think it has changed it big time, mate, I can tell you it has stopped me from going to hospital, that’s what I’m getting at. I no longer go to hospital, I don’t want to go to hospital. (PC2 Round 1)  … I think it improves the patient's understanding of how to manage their condition, plus giving them extra education. (GP6 Round 1) |
| **Suggestions**   - *Clarify patient access process* | *he was having problems with his breathing and food retention. And I rang up the clinic, and they said, “Oh, well, you have to get a referral.” Then the lady that looks after us, she said, “No, no, no, you don’t need to do that. I just ring your GP - your heart specialist, and he said, “Whenever you need to come in, you just come into the heart failure clinic and you can be looked after there (PC16 Round 2)  * I think we absolutely should do a nurse led patient hotline. So that we allow the patients when they are feeling off to call the nurses directly. They won’t call the doctors, but to call nurses directly and have the nurses be able to essentially triage over the phone (HS2/MG9 Round2) |

| **HealthPathways Website** | | |
| --- | --- | --- |
| **Implementation / delivery**   - *Continually updated part of the program delivery* - *Need for linked online forms* - *Easy process for GPs to follow correct treatment guidelines* - *Time required to write pathways* - *Maybe too much information* | * they're actually in the process of updating the diabetes pathways, we see it as our - part of what we need to do is to make sure that the information on the pathways is kept up to date and all the programs we've talked about there is information, and when we talk to GPs and we do case conferencing we say, "Go to the HealthPathways. What we've told you is there" (HS8/MG8 Round 1)  * our online forms aren’t quite there yet, but the idea is eventually we’ll be able to type our forms in and, again, put it onto that program.(AH2 Round 1)  * It’s actually a streamlined process for every GP in their LHD and they’re following in your guideline how to treat or how to follow the procedures in the right way. (CF4 Round 2)  *I spend a lot of time writing stuff for it and updating it and reviewing it, and I’m aware that other people have spent a much greater amount of time writing for it and updating it and reviewing it (HS2/MG9 Round 1)  *Lots of information But when there’s so much information, sometimes there’s information overload and people get lost. (HS7 Round 1) | |
| **Use**   - *Use increasing but still underutilised* - *Challenge of using in the consultation* - *Helpful information for GPs but takes time to explore* - *Potential for CF role in promoting* - *Used by practice staff and CFs* - *Used more by younger GPs* - *Used for referral pathways into LHD* - *Used for clinical updates and therapeutic guidelines* - *A resource that patients can also use* | *Yeah, yeah, I'm starting to use that more these days. I've put the little brochure on my wall where I keep my resources (GP6 Round 1)  *It is effective, and I use it all the time. (GP8 Round 2)  *I've hardly used it to be honest with you…I didn’t think it was – from my view I didn’t particularly find it very useful. (GP1 Round 1)  *well, HealthPathways is sort of gaining force or traction but it isn’t getting used as much as we’d hoped but I think.(GP5 Round 1)  *when I do use it, it's extremely useful but it's just having that time to access it and just remember it’s there…I've gone back and looked at it a few times after a patient's gone, but I should look at it while they were here. (GP9 Round 2)  *it’s a matter of them getting used to it and by taking the time out to explore the website and to know what’s on there, and for them again, it’s just another thing that adds onto their time… (CF3 Round 2)  *it’s still a work in progress, I do give that information to all my GPs the first time I meet with them and I constantly encourage them to use that, but I think it’s every now and then that I’ve come across a GP who is using it in their day-to-day practice (CF3 Round 2)  *I’ve used it once or twice, it is good. (GP13 Round 2)  * I may initiate looking at it to save time or to try and be efficient or to follow some of the things I could do, but a GP would always see – would follow-up a patient at the time. (PN3 Round 1)  *I tend to have it open as a quick reference for the patients here (PN4 Round 2)  * I utilise it predominantly to make sure that I’m on the right track… other times I will actually use HealthPathways, if I’m making a recommendation in management to the GPs (CF3 Round 1)  *I think that it is being used a lot by the GP registrars and the younger people …For established GPs I think occasionally they look it up for how to refer people into the LHD, and that is useful for them… (HS8/MG8 Round 1))  * I make use of it clinically and… occasionally reading through looking for things…. really valuable (MG5 Round 1))  *HealthPathways is very valuable. It's probably one of the main guidelines I use as well as therapeutic guidelines. Yeah, it's good in that it's got pretty much everything we need (GP12 Round 2)  *, I think even for patients, we’ve been giving them a website and they can have a look at the problems and they can look how to do things…There’s a lot of resources for patients as well. (CF4 Round 2) | |
| **Experience (positive)**   - *Helpful when dealing with complexity* | *as a GP I find it’s a valuable tool (MG5 Round 1))  * I had a patient with a range of difficult issues and it gets quite complex working out a path of action. The HealthPathways website was a great help. It covered topics that were relevant and helped me get his meds right. (GP12 Round 2) | |
| **Experience (negative)**   - *Difficult to navigate* | *Again, I know that it exists, and I’ve tried to look it up myself and there’s a few links and it’s not the most clear website, so it takes a bit of digging to find the right thing.(HS4 Round 1) | |
| **Satisfaction**   - *Information is updated* | *as a GP, I love it. (MG5 Round 1)  * It was changed a lot. So, a lot of information is now – has been uploaded and it’s really helpful. (PN1 Round 2)  *It’s up to date local information which is really important to me- not just stuff from somewhere overseas (GP8 Round 2) | |
| **Perceived value**   - *Better if simple* - *Internet access a barrier for some* - *Not fully localised to Australia but more local information is becoming available* - *Valuable for patient diagnosis and management* - *Helps GPs develop knowledge* - *Hospital staff endorse the information provided* - *Streamlines referral processes* | * from my view I didn’t particularly find it very useful. HealthPathways, what I’d find more useful is someone’s list. Something simple. (GP1 Round 1)  * like some GPs who are IT savvy would use it a lot more than others, whereas some would say it’s just too complex. I just want to know the answer rather than get into a computer website and reading through it. (CF3 Round 1)  * I think it’s a bit clunky. I think you need to look through things to be able to work out what's happening. But I would much rather have seen a published... rather than having to navigate a whole step plus process. I think it’s a little bit inefficient. (GP1 Round 1)  *… it’s not complete yet…some of the references are still in New Zealand (PN1 Round 1)  *I've been using that more often myself since starting with integrated care so that's been really good. I've got my sticker on my monitor so I’m able to log in and get all the access to that, and that's been really helpful, especially for the ones that have been localised already, so diabetes pathway for example, that's been really helpful. Yeah, that has been good actually; that's a very good resource. (GP6 Round 2)  *It’s been quite useful … a good resource for GPs even in terms of trying to work a patient up or trying to determine what investigations or management would be appropriate. I have found that very helpful. (GP4 Round 1)  * I'm actually finding it helpful, like the diabetes pathway and things like that is quite useful. I'm starting to use that much more, so I'm finding that that is beneficial as well for supporting GPs. (GP6 Round 1)  *I use HealthPathways all the time…I think there's a huge role for HealthPathways for everyone, junior and senior. Just so that you know what's out and available, and for clinical care, making decisions (GP7 Round 2)  *Quite good, it’s helped, it is so helpful for some information we can gather (GP11 Round 2)  * Improvement in my knowledge, especially through things like HealthPathways. So, not directly, but indirectly. I think that's been a big one. (GP2 Round 2)  *It’s a great website. Lots of information. (HS7 Round 1)  *I think it’s a great idea, I think there’s a lot of good information there, and I think it’s easy to navigate. I’m not that computer literate but I find it easy to navigate. (HN2 Round 1)  *knowing which clinic to refer to and the most streamlined processes for those clinics have been useful…having HealthPathways where each department will set down what they would like for a referral, I think it helps a lot (GP2 Round 2)  *It’s pretty good; especially it has the current referral. Let’s say I’m looking for an allied health in relation to podiatry it has a good list of the community available podiatrists… (PN6 Round 2) | |
| **Suggestions**   - *Localising* - *Promotion* - *Better if simplified* - *A reminder to use it* - *Should be expanded* | * So there are really good HealthPathways for cardiology and now respiratory, but they need to be better integrated into the conversations between specialists and GPs. (HS8/MG8 Round 1))  * we just need to get it localised as much as possible (GP2 Round 1)  * I don’t think enough GPs really know about it (GP2 Round 1)  * what I’d find more useful is someone’s list. Something simple. (GP1 Round 1)  *maybe we need to have some sort of a reminder or something to use it more often. You don't sort of think about it, you don't use it. And when you don't use it you sort of become unfamiliar with that. (GP9 Round 2)  *That's been really good. We'll want it to keep on growing, just to cover as much as possible. (GP2 Round 2) | |
| **Support payments for GPs** | | |
| **Implementation / delivery**   - *One off payment* - *Little disbursement to date* - *GPs unclear of the nature of the payment* - *Some compensation for the time required of a GP and practice staff* - *Still not rewarding quality practice* | | *the payment is very small and it’s a facility for change really, to say, if you invest a bit of time in this care plan we’ll give you some money. And that's not sustainable, it’s a one-off payment (MG2 Round 1))  *There’s very little that’s been paid out so far. I think at the moment we’ve paid out something like $5000 which is a pretty small amount across the practices. (MG5 Round 1))  …someone said it's a one-off sign-on payment to the patient and I think that - from what I can vaguely remember I think it would be fine, whatever the amount, but there is that extra time you need for that patient. (GP6 Round 1)  * I don’t think it relates very well to how much time gets put in. (GP5 Round 2)  *I know the practice gets something but we do so much more work-identifying and enrolling patients, preparing care plans follow ups and things like that (PN7 Round 2)  *There is a fair bit of work setting everything up for integrated care and it sort of covers that but it doesn’t cover all the other ongoing work (GP13 Round 2)  *it’s still a one off payment. That’s not a payment for overall care management or outcomes. So in some ways we’re just duplicating a faulty funding system by offering a one off payment. (MG6 Round 1) |
| **Use**   - *Conversation starter* - *Funds staff time for practice efficiencies* | | *it’s prompted and it’s driven some conversations. MG2 Round 1)  *it’s allowed us to spend more time getting our systems up to scratch in terms of patient records and care plans and setting up reminders for follow-up (GP14 Round 2) |
| **Experience (positive**)   - *Spending more time with patients* | | *we have always tried to provide holistic care for our patients and much of what we do is bulk billed so having that payment has allowed the practice to keep me more in the integrated care role and following up patients which I enjoy (PN5 Round 2) |

| **Experience (negative)**   - *GPs financially motivated* | * we've had multiple GPs sending patients and they know the criteria, but they just want to enrol because it's incentivised. I've had a patient ring the GP and the patient goes, “No I don't want to be part of the program,” and the GPs insisted “no you will”. (CF2 Round 1) |
| --- | --- |
| **Satisfaction** | *I’m happy that they recognise the extra work this generates and that some payment is needed (GP6 Round 2) |
| **Perceived value**   - *Does not cover work involved* - *Using other Medicare payments to cover costs* - *Not much of an incentive (token)* - *Helpful to cover additional time* - *Not a driving factor for some GPs* - *Creates greater collaboration between practice staff* | *if you just sit down and see how much work is involved, it’s not enough to do it. And if I've got to take time - my extra staff to start calling patients, that’s extra. (GP1 Round 1)  * I don’t think sometimes that’s good enough for GPs, they would like more or would expect more for their time (CF3 Round 2)  *it’s $75 twice a year …so I thought, ok let’s set it up …Now…I’m going to have to go through and check on these care plans every time, and if the doctors do the same thing, I’m not so sure now (PN7 Round 2)  *Is it adequate for us? Not from Doctor’s point of view, we could do a GP management plan, which generates $140 in how many minutes and what I’m trying to do is run off my integrated care program off the back of my GP management plans (PN5 Round 2)  * They’re a token, they're something but they're not sufficient to really be an incentive. They're a recognition of the work involved but not an adequate recompense in the sense. (MG3 Round 1)  *I’m not really sure they’re much of an incentive. They’re not much. I think it’s like $100, $150 per patient, per integrated care patient per year …A bit of a token. (GP5 Round 1)  * Well look, anything would help, particularly if our nurses, in the future, will be spending time doing it, and updating patients' care plans and reviewing them. (GP12 Round 2)  *I think it’s an adequate payment, yeah. (GP14 Round 2)  *quite often I do the Linked-EHR care plan after the patient's gone and – not necessarily on the same day. So that's still taking some time. So you kind of need to compensate the practice for that. But I do think the payments are adequate (PN4 Round 2)  *I get paid for work done, so yeah, I think that's important (GP2 Round 2)  * I received some payment, the practice will be given some portion of that for the patients I enrol. Yeah, I mean trying to, sort of, be a payment but – yeah. It’s not a driving - that payment is really not a driving factor for enrolment for me (GP2 Round 1)  * …incentives for the GPs they can then sit down with the practising nurse and then they can read through the Linked-EHR … It’s an incentive for the doctors putting extra time for their patients (CF4 Round 2) |
| **Suggestions** | * The one off is fine for getting things up and running but it’s the ongoing and follow-up that takes time which we need to cover-perhaps something related to that could be arranged for those patients that need more work (GP10 Round 2) |
| **Patient Centred Medical Home (PCMH)** | |
| **Implementation / delivery**   - *PHN initiative* - *Alignment of LHD and PHN* - *Registrar teaching re PCMH* - *Awareness building* - *Similarity with Integrated care will assist PCMH transition* - *Funding seen as a barrier* | * The LHD's involvement in that is much more peripheral…it is being completely driven by the PHN. (MG3 Round 1)  *the LHD is aligned with the idea that the general practitioner is the patient’s medical home and that’s where the complete data should reside and that’s where the care planning should primarily be done and that the role of the specialist and others, is to support the patient and the GP in the community (MG3 Round 2)  * it fits with the whole concept of having everybody that cares for a patient all on the same page…for a subset of my patients, it definitely aligns well with the PCMH models where having a care plan which is viewable by everyone is great… (GP2 Round 2)  * I’m a registrar so we did get a bit of teaching through WentWest about that and had a few discussions with some other colleagues about the patient centred medical home. (GP4 Round 1)  *And they hear health care home and they hear the things in the media, and stuff like that. So it’s not new anymore. People have heard of it. (HN4 Round 2)  * The doctors are already aware of integrated care/patients at a medical home, so you incorporate very similar themes. It’s helped bring people on board quicker because they’re already aware of what it’s all about. (GP5 Round 2  *.. quite a few practices…are going to be working with the patient centred medical home model…most of the practices that are transitioning are the ones that have been working well with integrative care (CF3 Round 2)  * We have been thinking about heading that way in terms of the patient centred medical home. What has the Integrated Care program done to facilitate that? I guess in a way it has helped us with that thinking or with that mentality of looking at patients in a more holistic kind of approach (GP4 Round 2)  * this practice is really working towards that so we have kind of got that team based approach (GP6 Round 2)  * I was enrolling patients and empanelling them for different doctors. That’s been a good move from the Integrated Care Program, which is helped with PCMH model overall as well (GP2 Round 2)  * There's not enough funding there. And it’s too restricted to just a certain cohort of patients. So we are just pressing on with developing a PCMH-style practice, with the current funding model, and just figuring out how to best use what we have, so that we can fund that style of care. (GP7 Round 2) |
| **Use** | * |
| **Experience (positive)** | * |
| **Experience (negative)** | * |
| **Satisfaction** | * |
| **Perceived value**   - *Aspirational* - *PCMH sign of innovation* - *Multidisciplinary* - *Holistic care* - *Patientcare in the community and hospital avoidance* - *Reduces risk of adverse events related to multiple independent providers* | * I liked the idea, yeah, but I'm not sure yet until we get everything in place that it has been achieved, but yes, it certainly is an excellent goal, yeah. (HN4 Round 1)  *I think the patient centred medical home … was a significant influence on the demonstrator and us getting the demonstrator … already ahead of the game moving towards the patient centred medical home (MG6 Round 2)  *a good idea because in a sense that’s also somewhat kind of like a multidisciplinary approach, that they see the nurse, they see other people before they see the doctor and it’s everything kind of based around, holistic care for the patient. (GP4 Round 1)  * it really helps to optimise the patient care and share the care around allied health and the team members, and it helps for the patients to understand that they have got a team around them and not just one individual provider (GP6 Round 2)  * the concept is about providing as much care as you can for the patient and the community. So it’s not about the home, it’s about the community, how you’ll use the community to manage the patient so that they don’t need to go to hospital. (MG2 Round 1)  * I think one of the really big challenges in general practice is people going into multiple practitioners and having lots of things done and I think having a service where patients get their health care and we remove that risk of drug interactions, of different treatments … (MG5 Round 1) |
| **Suggestions**   - *Integrate hospital specialists with community* - *Funding essential* | * it’s about managing the patient with what's available in the community. But with truly integrated care then, yes, there is a role somewhere in the future to have the specialists out in the community, be it through technology like case conference and virtually through Skype or whatever. (MG2 Round 1)  … it just needs to be properly funded so people can put the proper resources into it. (MG5 Round1)  * you can see that same attitude coming through with the health care home stuff from the Commonwealth. They’re talking about shifting from volume to value, but really what they’ve done is to offer greater flexibility in utilising exactly the same number of dollars, which is hardly a big incentive (MG3 Round 2) |
| **Communication with other (Non-WSICP) Services** | |
| **Implementation / delivery**   - *Accessing existing contacts and networks* - *Linking into external services including non-health services* - *Limited integration and co-location with community sector* | *Outside, community nursing, I worked hard when I first took on this role so we had a good foot in the door with them as well so integrated care could link in with that, and they are aware of integrated care…(HN4 Round 1)  * getting quite a good communication going between the community nurses and the heart failure service, so that’s continuing… (HN4 Round 2)  *so now all referrals to My Aged Care, so, for home mods, equipment, any community services such as podiatry, or showering assistance, or nursing care, or anything, has to go through My Aged Care (AH4 Round 1)  * Now that we’ve got My Aged Care, if they’re over 65 it’s a different form to refer (CF3 Round 1)  * we've linked up GPs with their optometrists and we're actually standardising the referral from a GP to a preferred optometrist and then getting information from the optometrist back to the GP and anyone identified with diabetic eye disease goes to the secondary centre and can get rapidly triaged into the hospital (HS8/MG8 Round1)  *doing a bit of that work in diabetes, it’s about urban design, transport, food supply and physical activity (HS8/MG8 Round 1) |
| **Use**   - *Growing relationships with private providers* - *Building multidisciplinary expertise in general practices* - *Connections with other Government and NGO organisations* - *WSICP facilitates awareness of non-WSICP providers* - *Referrals are still mostly internal* | *we’ve got linked in with a private heart scan at Merrylands which has got a really good scanner for coronary CT angiograms. … we have a really good relationship there as well (HN2 Round 1)  *… I’ve been in contact with community dieticians that I haven’t been in contact with before and I think that will have benefits in both of my roles. (AH6 Round 1)  *In terms of the other community OTs that you are referring to, or trying to get that information from, I find that allied health are pretty good. You – like, if I call they may get back to you later in the day (AH4 Round 1)  * generally it would be the community OTs or the PACC OTs or the OTs that work with the home mod service that’s really good it just takes a phone call if you’re not sure what to do and that’s all really quite easy (AH4 Round 2)  *We’re starting to use a pharmacist in our practice… would be so helpful … because the pharmacists have so much knowledge that they could be helping us with and saving us problems and preventing medical issues occurring (GP5 Round 1)  * I think not just verbal but written down….My Aged Care. They leave a folder there, all the PACC service leave a folder… and you can look up their care support person, or whatever, and then you can ring them (AH4 Round 1)  *Some of the Community Services – we do interact with things like PACC Services and sort of all those existing ones that we’ve been involved with, but sometimes patients already have some links to community services where we can just let them know that the patient’s going back (HS1/MG7 Round 1)  * the main ones I talk to are community health, mainly OTs, Home Modification Service, My Aged Care, Meals on Wheels sometimes, and all of those community agencies, and I think, communication wise, they’re very good, but if you actually go through the central system, like My Aged Care is still breaking down a tad. (AH4 Round 1)  * Other people at the Mt Druitt Community Health, not with integrated care, but the child family nurse, and they get back you by phone and in writing usually, which is really good, so you get the verbal and you can talk to them in person as well (GP6 Round 2)  * we do use Connecting Care is another one (AH1 Round 2)  *I have come to know more health care providers through the program. Prior to the program I never bothered to know the community nurses as much. I do refer to them, but won’t have taken an extra interest in them. (HN3 Round 1)  * I’ve communicated a lot with the podiatrists, because I’ve been searching some of the really good ones (PN6 Round 2)  *fair comment to say is that I haven’t been involved in a lot of the processes outside the hospital, and, I think, because we’re getting such great links with our in-hospital connections, if I’ve got a problem, I tend to go to my in-hospital link, because it’s easy… (AH3 Round 1) |
| **Experience (positive)**   - *Allied health services accessed for home modifications* | *The hospital organised things like rails for the shower and front steps and connected us with home care to help me with cleaning-that happened really quickly and I feel [husband] is much safer now. (PC22 Round 2)  *My doctor organised a community nurse who calls in regularly-because I can’t get out easily with my COPD-she checks up on me and lets me know about things I can do and who to contact for transport and things like that-she always spends time with me (PC2 Round 2)  *we’ve had patients that have needed assistance from the Health One team as well, so the integrated care coordinators have been able to bring that in and get that liaising as well, so it's all kind of streamlined in kind of one place, so that's been really good (GP6 Round 2) |
| **Experience (negative)**   - *Greater community integration needed* - *Feedback from external providers can be slow* - *Communication not well connected across all providers* | *You would have positions that have a component which is working in a community hub, community practice or both, that needs to be built into the system at this stage and absolutely isn’t (MG3 Round 1)  *I’m aware of things like PACC, and My Aged Care and various places… but in terms of getting feedback from them and knowing what's happened to my patients between when I last saw them and when I next saw them, I don’t tend to get a good sense of that, I just feel like the communication is pretty messy (AH6 Round 1)  *Other allied health that are in the community, always a little bit more tricky, but when you are kind of waiting for that letter to come back to find out what's happened or things are a bit slow (CF2 Round 2)  *We send all the information through with a referral… doesn’t seem to be getting to the home mods service. ..it just lengthens the process of – before the person can get the rails…(AH4 Round 1)  *the problem is that while we’ve got good systems in place, so primary healthcare, the specialist healthcare, a lot of patient and community systems, they don’t talk to each other and they don’t have good forms of communication or referral in and out.(HS3 Round 1) |
| **Satisfaction**   - *Collaboration with other hospitals* | * I try to have some connection with Concord as well. But it’s good to collaborate with each place see what we’re doing, get ideas from each other (HS7 Round 1) |
| **Perceived value**   - *Engagement with community benefits WSICP roles* - *Time to build awareness of other services* - *Some services do not have capacity* | *Yeah, look, I’ve been in contact with community dieticians that I haven’t been in contact with before and I think that will have benefits in both of my roles [dietician and educator]. (AH6 Round 1)  *we deal with Heartscan at Merrylands and they’re fantastic , and there’s an agreement there with Medicare so the patient doesn’t pay (HN2 Round 2)  *Just having the time to spend on making some phone calls, talking to some dieticians in private practice in the area, looking at community services and how the referral systems work has given me a better understanding of what's out there (AH6 Round 1)  *I'm more aware they're out there. That's probably the thing I'm aware what the services that are available out there for the patients with different things (PN2 Round 1)  * NDIS is a mess, a big mess, and, it’s that – I suppose it’s just been rolled out so it will get better but there’s a huge time delay with people being able to be assessed (CF2 Round 2) |
| **Suggestions**   - *Prevent fragmented care* - *Share information* | *we’ve decided to give our patients the little card that says they come to us, and if they come to a different practice, could you please send a copy of any results, or a copy of your medical notes, so we can follow on their care. I don’t know if that will work or not, but it’s that fragmentation you need to address (GP7 Round 2)  * just in terms of whether it’s in the community or whether its, home mods or whatever , or someone working in one of the respiratory clinics it would be good if we could all get the same information (AH4 Round 2) |
